# Enthalpic incompatibility between two steric stabilizer blocks provides control over the vesicle size distribution during polymerization-induced self-assembly in aqueous media†
†Electronic supplementary information (ESI) available: Experimental details, copolymer characterization techniques, supporting figures: assigned ^1^H NMR spectra, GPC curves, tabulated GPC data, DLS phase diagram for [*x***PEG_113_** + (1 – *x*) **PEG_45_**] – **PHPMA_400_** nano-objects, TEM images for [*x***PEG_113_** + (1 – *x*) **PEG_45_**] – **PHPMA_400_** vesicles and tabulated ammonium sulfate solution viscosities. See DOI: 10.1039/d0sc01320j


**DOI:** 10.1039/d0sc01320j

**Published:** 2020-06-29

**Authors:** Deborah L. Beattie, Oleksandr O. Mykhaylyk, Steven P. Armes

**Affiliations:** a Department of Chemistry , University of Sheffield , Dainton Building, Brook Hill , Sheffield , South Yorkshire, S3 7HF , UK . Email: s.p.armes@shef.ac.uk ; Email: o.mykhaylyk@sheffield.ac.uk

## Abstract

Over the past two decades, block copolymer vesicles have been widely used by many research groups to encapsulate small molecule drugs, genetic material, nanoparticles or enzymes. They have also been used to design examples of autonomous self-propelled nanoparticles. Traditionally, such vesicles are prepared *via* post-polymerization processing using a water-miscible co-solvent such as DMF or THF. However, such protocols are invariably conducted in dilute solution, which is a significant disadvantage. In addition, the vesicle size distribution is often quite broad, whereas aqueous dispersions of relatively small vesicles with narrow size distributions are highly desirable for potential biomedical applications. Alternatively, concentrated dispersions of block copolymer vesicles can be directly prepared *via* polymerization-induced self-assembly (PISA). Moreover, using a binary mixture of a relatively long and a relatively short steric stabilizer block enables the convenient PISA synthesis of relatively small vesicles with reasonably narrow size distributions in alcoholic media (C. Gonzato *et al.*, *JACS*, 2014, **136**, 11100–11106). Unfortunately, this approach has not yet been demonstrated for aqueous media, which would be much more attractive for commercial applications. Herein we show that this important technical objective can be achieved by judicious use of two chemically distinct, enthalpically incompatible steric stabilizer blocks, which ensures the desired microphase separation across the vesicle membrane. This leads to the formation of well-defined vesicles of around 200 nm diameter (size polydispersity = 13–16%) in aqueous media at 10% w/w solids as judged by transmission electron microscopy, dynamic light scattering and small-angle X-ray scattering.

## Abstract 2

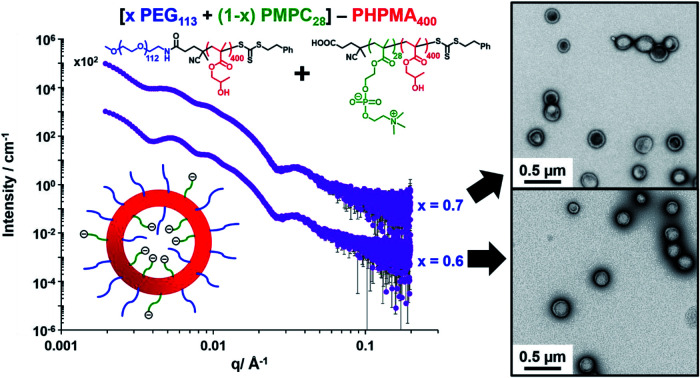
SAXS studies confirm that a judicious binary mixture of enthalpically incompatible steric stabilizer blocks enables the synthesis of relatively small, well-defined vesicles *via* polymerization-induced self-assembly in aqueous media.

## Introduction

Over the past decade or so, polymerization-induced self-assembly (PISA) has become widely recognized as a highly efficient and versatile technique for the rational synthesis of a wide range of block copolymer nano-objects in concentrated solution.^1^,^2^ Systematic variation of the relative volume fractions of the solvophilic and solvophobic blocks allows convenient access to sterically-stabilized spheres, worms and vesicles using many different monomers.^3^–^11^ In principle, various types of controlled/living polymerization techniques can be used for such PISA syntheses but reversible addition–fragmentation chain transfer (RAFT) polymerization is most commonly reported in the literature.^12^

RAFT-mediated PISA can be conducted in a wide range of solvents.^13^–^20^ In practice, water is the most cost-effective, environmentally-friendly and is also best suited for potential biomedical applications. Of particular relevance to the present study, RAFT aqueous dispersion polymerization^2^,^21^,^22^ involves chain extension of a water-soluble homopolymer with a water-miscible monomer such as 2-hydroxypropyl methacrylate (HPMA),^23^–^26^ diacetone acrylamide (DAAM),^27^–^29^ *N*-isopropyl acrylamide (NIPAM)^30^ or 2-methoxyethyl acrylate (MEA).^31^,^32^ This growing second block eventually becomes insoluble at some critical degree of polymerization (DP), thus triggering *in situ* self-assembly to form diblock copolymer nanoparticles.

Two of the earliest RAFT aqueous dispersion polymerization formulations involved the use of poly(glycerol monomethacrylate) (PGMA) or poly(2-(methacryloyloxy)ethyl phosphorylcholine) (PMPC) as the water-soluble precursor block to grow a hydrophobic structure-directing PHPMA block.^23^,^33^–^36^ Numerous studies have indicated that PGMA and PMPC are highly attractive building blocks for the rational design of nanobiomaterials owing to their proven biocompatibility.^37^–^47^ Similarly, poly(ethylene glycol) (PEG) is a well-known biocompatible polymer that can be utilized as a hydrophilic steric stabilizer.^6^,^26^,^48^–^55^ In particular, PISA enables PEG-PHPMA thermoresponsive worms and vesicles to be readily prepared directly in aqueous solution.^8^,^48^,^49^,^54^ The worms form soft, free-standing physical gels that can be used for 3D cell culture but the vesicles tend to be relatively large and rather polydisperse in terms of their size distribution.^38^,^48^,^55^–^58^ Controlling the particle size distribution of block copolymer vesicles is an important and long-standing scientific problem, with various ingenious strategies being reported in the literature.^59^–^69^

For example, Luo and Eisenberg reported that judicious use of a binary mixture of two AB diblock copolymers enabled the preparation of low-dispersity vesicles.^61^,^70^,^71^ More specifically, using a relatively long and a relatively short poly(acrylic acid) (PAA) stabilizer block in combination with a common hydrophobic polystyrene (PS) block led to spatial segregation across the vesicle membrane. The longer PAA chains were preferentially expressed at the outer leaflet, while the shorter chains were located within the more sterically-congested inner leaflet, with this configuration lowering the free energy for the vesicle morphology. Luo and Eisenberg later demonstrated that the same principle was also applicable for spatial segregation of two chemically distinct corona blocks – PAA and poly(4-vinylpyridine) – across a PS membrane.^72^ However, these studies utilized a traditional post-polymerization processing route that required a water-miscible co-solvent and a relatively low copolymer concentration. Thus, it does not provide a scalable route to well-defined vesicles. Subsequently, Gonzato *et al.* showed that this ‘binary mixture of steric stabilizers’ concept was also valid for PISA formulations by demonstrating the synthesis of relatively small, low polydispersity vesicles using RAFT alcoholic dispersion polymerization.^73^ Both dynamic light scattering and small-angle X-ray scattering (SAXS) studies indicated a significant reduction in the width of the vesicle size distribution. However, as far as we are aware, this approach has not yet been reported for any aqueous PISA formulations. This omission is perhaps surprising given the strong interest in using block copolymer vesicles for a wide range of biomedical applications, including intracellular delivery of drugs,^74^–^76^ genes^77^–^79^ or antibodies,^80^ antibacterial agents^81^,^82^ for autonomous locomotion^83^ or chemotaxis^84^ and for encapsulation of therapeutic enzymes.^51^,^57^,^58^,^85^

**Scheme 1:**
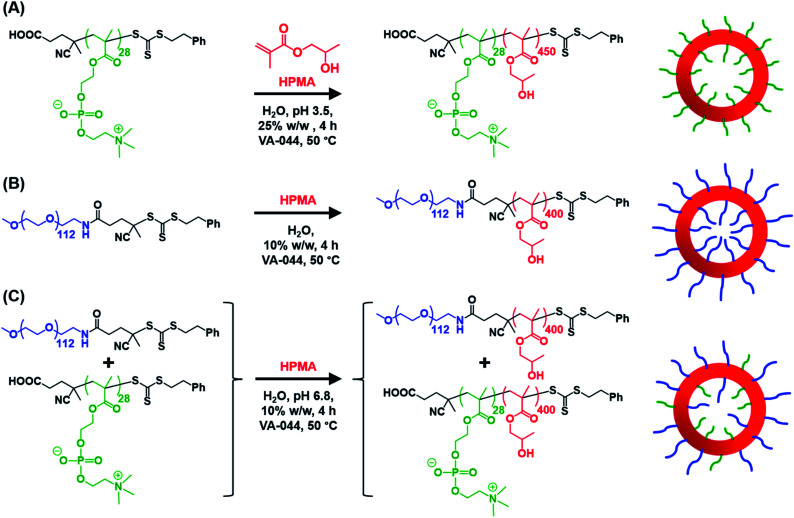
Schematic representation of the RAFT aqueous dispersion polymerization of HPMA at 50 °C to produce (A) 
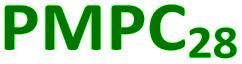
–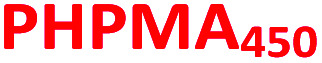 vesicles at 25% w/w solids; (B) 
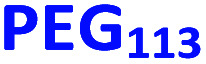
–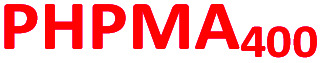 vesicles at 10% w/w solids and (C) [*x*
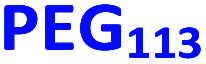
 + (1 – *x*) 
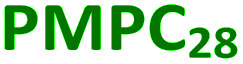
] – 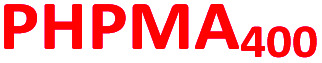 vesicles at 10% w/w solids.

Herein, we report the rational synthesis of relatively small diblock copolymer vesicles with narrow size distributions directly at 10% w/w solids using an aqueous PISA formulation. This is achieved by employing two *chemically dissimilar* stabilizer blocks of differing degrees of polymerization (see Scheme 1). These two stabilizers are non-ionic PEG and zwitterionic PMPC, whose mutual enthalpic incompatibility has been previously reported by Blanazs and co-workers.^86^ We use SAXS analysis to demonstrate that using such a binary mixture of enthalpically incompatible stabilizers is essential to exert the desired control over the vesicle size distribution during aqueous PISA. In contrast, a binary mixture of *chemically identical* stabilizers appears to reduce the breadth of the vesicle size distribution but only produces relatively large vesicles, which are considered to be less useful for many potential biomedical application.

## Results and discussion

### Targeting the vesicle morphology

It is well known in the block copolymer literature that asymmetric diblock copolymer compositions must be targeted to access vesicle phase space. This was originally established by Eisenberg and co-workers using traditional post-polymerization processing *via* a solvent switch in dilute solution^87^,^88^ but it is equally valid for PISA syntheses.^33^,^35^ This design rule can be explained in terms of the geometric packing parameter *P* introduced by Israelachvili and co-workers to account for surfactant self-assembly,^89^ which has been subsequently validated for the self-assembly of amphiphilic diblock copolymers.^90^ If *P* is greater than 0.50, then vesicles are favored unless there are other constraints, in which case kinetically-trapped spheres may be formed.^91^

### Vesicle synthesis strategy

Informed by this design rule, Sugihara and co-workers reported that targeting **PMPC_25_**–**PHPMA_400_***via* aqueous PISA at 25% w/w solids produced a rather polydisperse vesicular morphology at 70 °C.^34^ In the present study, a closely-related aqueous PISA formulation was used to produce **PMPC_28_**–**PHPMA_450_** vesicles (Scheme 1a) by targeting the same relatively high copolymer concentration and a slightly longer structure-directing PHPMA block. A lower reaction temperature of 50 °C was also employed but the most important difference is that a carboxylic acid-functionalized RAFT agent was used to prepare the PMPC precursor block. This was a deliberate choice because this ionizable end-group is known to influence the electrophoretic behavior of block copolymer nano-objects,^92^,^93^ which was expected to aid discrimination between the three types of vesicles shown in Scheme 1. To ensure the formation of pure **PMPC_28_**–**PHPMA_450_** vesicles, PISA syntheses were conducted at low pH to prevent end-group ionization reducing the packing parameter, which would inevitably lead to kinetically-trapped spheres.^92^,^93^ The HPMA polymerization proceeded to more than 99% conversion within 4 h and GPC studies (refractive index detector, 3 : 1 chloroform/methanol eluent) indicated a relatively high blocking efficiency (see Fig. S2†). A relatively broad molecular weight distribution was obtained (see Table S1†) but this was not unexpected given the known contamination of the HPMA monomer with a dimethacrylate impurity, which inevitably leads to branching when targeting a relatively high degree of polymerization (DP).^33^ Indeed, a relatively high dispersity was also reported by Sugihara *et al.*^34^

Similarly, the **PEG_113_**–**PHPMA_400_** formulation outlined in Scheme 1b has already been reported by Warren *et al.*, who obtained relatively large polydisperse vesicles when conducting such aqueous PISA syntheses at 10% w/w solids.^48^ In the present study, a HPMA conversion of more than 99% was achieved within 4 h at 50 °C, while 3 : 1 chloroform/methanol GPC studies indicated an *M*_n_ of 45 400 g mol^–1^ and an *M*_w_/*M*_n_ of 1.45 for the **PEG_113_**–**PHPMA_400_** chains (see Fig. S2 and Table S1†). These GPC data are similar to that reported by Warren and co-workers.^48^

Bearing in mind the earlier PISA studies by Gonzato *et al.*,^73^ we hypothesized that using a judicious binary mixture of a relatively long **PEG_113_** stabilizer and a relatively short **PEG_45_** stabilizer while targeting a sufficiently long structure-directing PHPMA block should yield small vesicles with a relatively narrow size distribution. However, this strategy proved fruitless, as summarized in Fig. S3 and S4.† Regardless of the **PEG_113_** mole fraction employed, only relatively large and/or polydisperse vesicles (or even less well-defined structures) could be obtained.^94^ To address this unexpected problem, we speculated that a binary mixture of a pair of *chemically dissimilar* stabilizer blocks should enhance the apparently weak segregation of the long and short chains across the vesicle membrane and ideally also simultaneously reduce the vesicle size. According to Blanazs and co-workers, **PEG_114_** and **PMPC_50_** homopolymers are sufficiently enthalpically incompatible to form an aqueous biphasic solution, while **PEG_114_**–**PMPC_23_** diblock copolymers forms a range of structures exhibiting long-range order in concentrated aqueous solution.^86^ Thus we explored the synthesis of hybrid vesicles using the PISA formulation outlined in Scheme 1c. Based on the prior studies by Eisenberg *et al.*,^72^ this strategy should result in the relatively long **PEG_113_** stabilizer chains being preferentially expressed at the outer leaflet of the vesicle membrane, while the relatively short **PMPC_28_** chains should be located within the vesicle lumen. The **PEG_113_** mole fraction was systematically varied when preparing a series of [*x***PEG_113_** + (1 – *x*) **PMPC_28_**] – **PHPMA_400_** nano-objects. Given the well-known tendency for the **PEG_113_** stabilizer block to produce either oligolamellar vesicles or insoluble precipitates when targeting longer PHPMA blocks at high solids,^48^ these PISA syntheses were conducted at 10% w/w solids to ensure formation of unilamellar vesicles. More than 99% HPMA conversion was achieved within 4 h and a high blocking efficiency was obtained for each of these aqueous PISA syntheses (see Fig. S2 and Table S1†).

### Kinetic studies of the synthesis of [*x***PEG_113_** + (1 – *x*) **PMPC_28_**] – **PHPMA_400_** diblock copolymer nanoparticles

For the synthesis of small vesicles with a relatively narrow size distribution, Gonzato *et al.* employed a binary mixture of a relatively long and a relatively short poly(methacrylic acid) (PMAA) precursor for the RAFT dispersion polymerization of benzyl methacrylate (BzMA) in ethanol.^73^ Both stabilizer blocks were prepared using the same trithiocarbonate-based RAFT agent and it was implicitly assumed that the structure-directing poly(benzyl methacrylate) (PBzMA) chains grown from each of these PMAA stabilizers would have the same mean DP. However, this may not necessarily be the case. This is because the critical PBzMA DP required for micellar nucleation actually depends on the DP of the PMAA stabilizer block. Thus, nucleation should commence at a significantly lower critical PBzMA DP when using the shorter PMAA_62_ block compared to when utilising the PMAA_171_ block. In principle, this could be important, because the nascent nuclei quickly become swollen with unreacted BzMA and the ensuing high local monomer concentration leads to a substantial increase in the rate of polymerization.^12^,^35^,^95^ Thus if micellar nucleation is delayed for the PMAA_171_ block, the PBzMA chains grown from this precursor are likely to be shorter than those grown from the PMAA_62_ block. Moreover, this suggests that the membranes of the resulting vesicles might comprise a bimodal distribution of PBzMA chain lengths. However, it is perhaps also worth bearing in mind that block copolymer self-assembly can be remarkably tolerant of dispersity effects.^70^,^96^–^98^

**Fig. 1:**
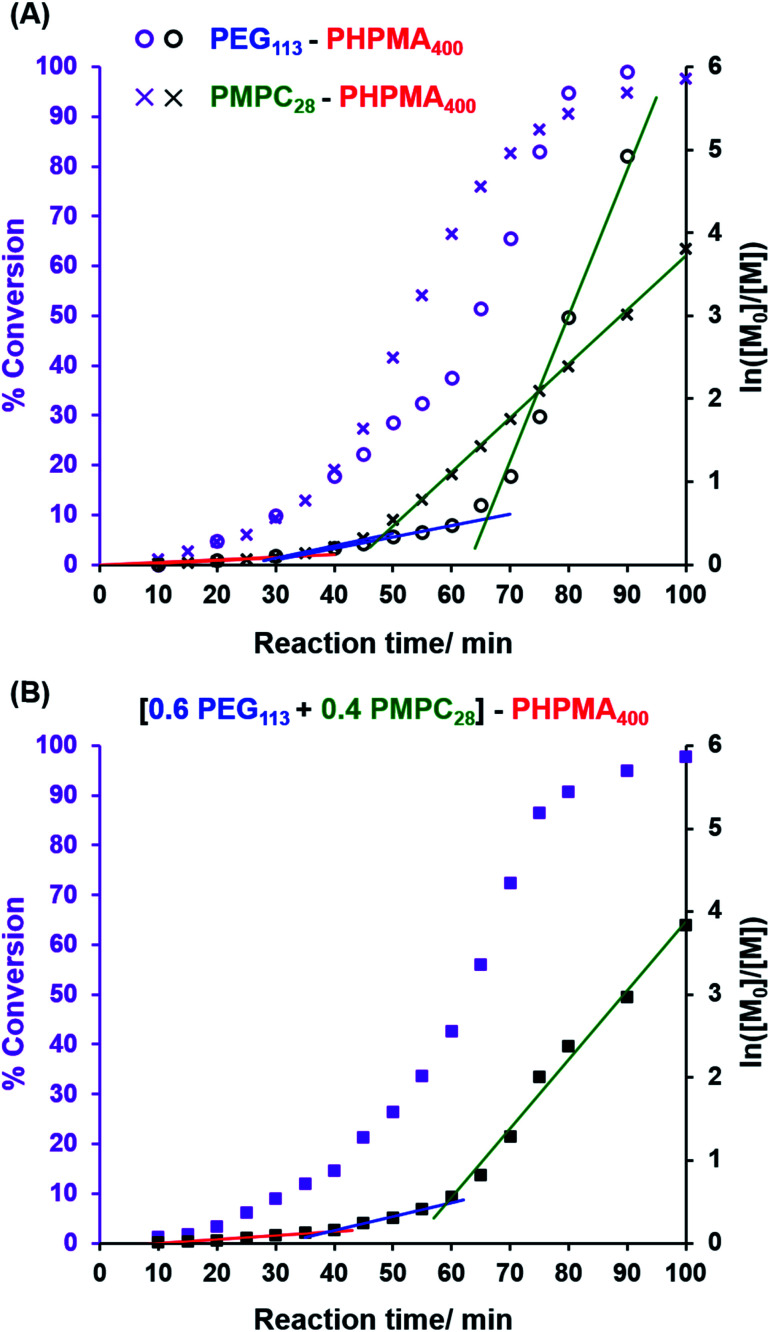
Conversion *vs.* time curves and the corresponding semilogarithmic plots obtained from ^1^H NMR studies of the RAFT aqueous dispersion polymerization of HPMA at 50 °C when targeting a PHPMA DP of 400 at approximately 10% w/w solids. (A) Using either a **PEG_113_** precursor or a **PMPC_28_** precursor as the steric stabilizer block. (B) Using a binary mixture of steric stabilizer blocks comprising 0.60 **PEG_113_** and 0.40 **PMPC_28_**.

In the present study, we undertook kinetic experiments in order to assess to what extent the critical DP required for micellar nucleation differs for the **PMPC_28_** and **PEG_113_** precursors. The conversion *vs.* time curves and corresponding semilogarithmic plots obtained from ^1^H NMR studies are shown in Fig. 1A when using each of these stabilizer blocks in turn for the RAFT aqueous dispersion polymerization of HPMA. As previously reported by Cornel and co-workers,^99^ three distinct stages are observed for each polymerization, with the second inflection point being assigned to micellar nucleation. As expected, this event occurs at a significantly earlier stage when using the **PMPC_28_** precursor. In this case, an eight-fold increase in the rate of polymerization of HPMA occurs after 48 min, which corresponds to an instantaneous conversion of 30% and hence a critical PHPMA DP of 120. In contrast, a rate acceleration is not observed until 66 min when using the **PEG_113_** precursor, which corresponds to an instantaneous conversion of 42% and a critical PHPMA DP of 168. Interestingly, a 31-fold rate enhancement is observed in this case, which might be expected to mitigate the anticipated bimodal distribution of PHPMA chain lengths when using a binary mixture of **PMPC_28_** and **PEG_113_** stabilizers. Indeed, GPC analyses of nominal binary mixtures of **PEG_113_**–**PHPMA_400_** plus **PMPC_28_**–**PHPMA_400_** do not provide any evidence for a bimodal molecular weight distribution (see Fig. S2†).

The kinetic data obtained when employing a binary mixture of 0.60 **PEG_113_** and 0.40 **PMPC_28_** stabilizer blocks are shown in Fig. 1B. Perhaps surprisingly, only a *single* micellar nucleation event is observed for this latter formulation: a nine-fold rate enhancement occurs after 59 min, which corresponds to 38% conversion and hence a critical PHPMA DP of 152. Thus, micellar nucleation occurs at a time point (and a critical PHPMA DP) that is intermediate between those observed in Fig. 1A. This suggests that these nascent micelles actually comprise a binary mixture of **PEG_113_**–**PHPMA_152_** and **PMPC_28_**–**PHPMA_152_** chains owing to entropic mixing of the growing amphiphilic copolymer chains during PISA. This interpretation is consistent with the corresponding aqueous electrophoresis data (see later) obtained for the three types of vesicles described in Scheme 1. Thus, our initial concern regarding the potential problem of a bimodal distribution of PHPMA chain lengths being generated during such PISA syntheses appears to be unfounded.

### Structural characterization of [*x***PEG_113_** + (1 – *x*) **PMPC_28_**] – **PHPMA_400_** diblock copolymer nano-objects

**Fig. 2:**
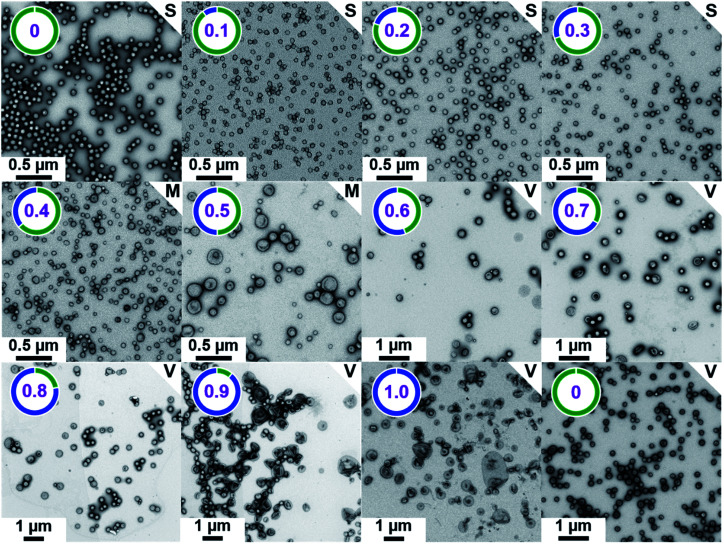
Representative TEM images recorded for [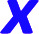 
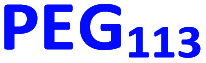
 + (1 – 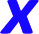) 
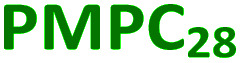
] – **PHPMA_400_** diblock copolymer nano-objects prepared at 10% w/w solids *via* RAFT aqueous dispersion polymerization of HPMA at 50 °C while systematically varying the mole fraction (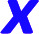) of the 
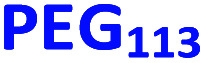
 steric stabilizer block from 0.0 to 1.0. The number in purple denotes 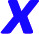, while S indicates spheres, M indicates a mixed phase of spheres and vesicles, and V indicates vesicles. Finally, 
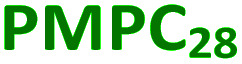
–**PHPMA_450_** vesicles (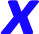 = 0) prepared at 25% w/w solids are also included as a reference.

**Fig. 3:**
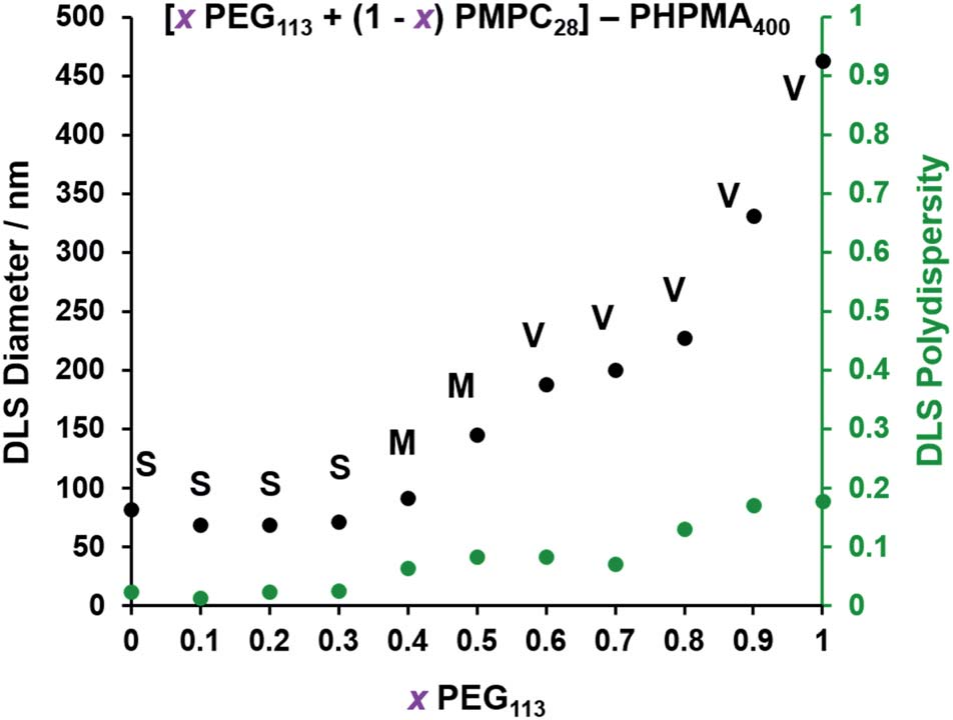
Effect of systematically varying the **PEG_113_** mole fraction on the particle size distributions of the resulting [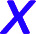 **PEG_113_** + (1 – 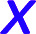) **PMPC_28_**] – **PHPMA_400_** diblock copolymer nano-objects as judged by DLS. Intensity-average diameters and polydispersities were determined for 0.1% w/w aqueous dispersions diluted from the as-synthesized 10% w/w dispersions using deionized water. S indicates spheres, M indicates a mixed phase of spheres and vesicles, and V indicates that vesicles were the predominant morphology.

The TEM images shown in Fig. 2 were used to assign the predominant copolymer morphology and this information was combined with DLS data to construct Fig. 3. This phase diagram is strikingly similar to that reported by Gonzato *et al.*,^73^ but differs markedly from that shown in Fig. S3† for the synthesis of [*x***PEG_113_** + (1 – *x*) **PEG_45_**] – **PHPMA_400_** nano-objects.

**Fig. 4:**
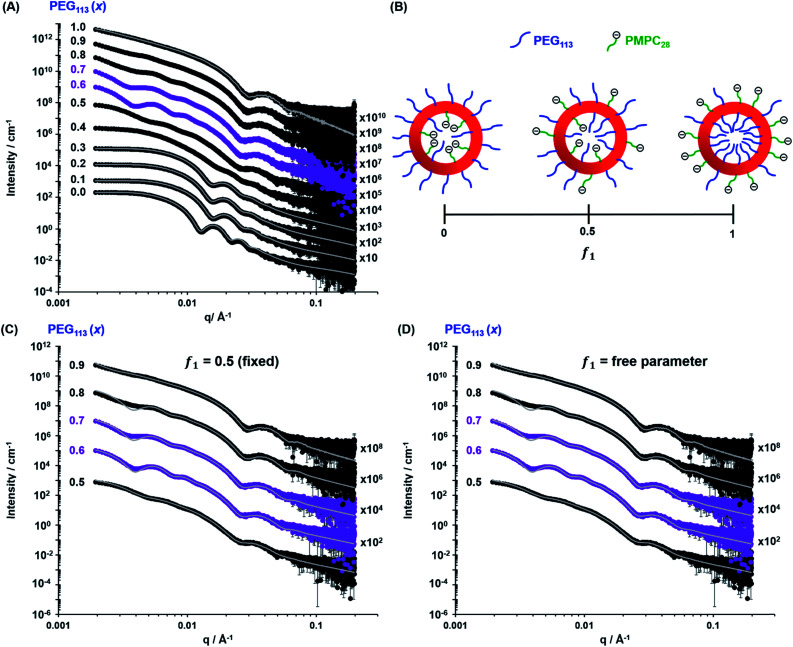
(A) Experimental SAXS patterns (symbols) and corresponding data fits (grey lines) obtained for 1.0% w/w aqueous dispersions of [*x***PEG_113_** + (1 – *x*) **PMPC_28_**] – **PHPMA_400_** nano-objects originally prepared at 10% w/w solids, where the mole fraction (*x*) ranges from 0.0 (**PMPC_28_**–**PHPMA_400_**) to 1.0 (**PEG_113_**–**PHPMA_400_**). Scattering curves obtained for nano-objects prepared using a PEG mole fraction of 0.1–0.3 could be satisfactorily fitted using the blob-modified spherical micelle model, while the scattering patterns obtained for **PMPC_28_**–**PHPMA_400_** spheres were fitted using a well-known spherical micelle model.^100^ A well-established vesicle model^101^ was employed to fit the scattering pattern recorded for **PEG_113_**–**PHPMA_400_** vesicles obtained when *x* = 1.0. (B) Schematic representation of the segregation fitting parameter *f*_1_, which indicates the mole fraction of **PMPC_28_** chains occupying the outer leaflet of the vesicles. (C) Experimental SAXS patterns [same symbols as shown in (A)] recorded for the sub-set of [*x***PEG_113_** + (1 – *x*) **PMPC_28_**] – **PHPMA_400_** vesicles obtained for *x* = 0.50–0.90. These patterns were fitted using a refined vesicle model that takes account of the binary mixture of diblock copolymer chains [eqn (16)] using a fixed segregation parameter *f*_1_ of 0.50 (which corresponds to a purely statistical mixture of PEG and PMPC stabilizer chains occupying the outer corona). The grey lines indicate the data fits obtained using this more sophisticated scattering model. In contrast, (D) shows the data fits obtained when *f*_1_ is allowed to vary as a free parameter.

For PISA syntheses conducted using relatively low levels of **PEG_113_** stabilizer (*i.e.* for either pure **PMPC_28_** or **PMPC_28_**-rich formulations), only kinetically-trapped spheres could be obtained at 10% w/w solids. Moreover, using a **PEG_113_** mole fraction of either 0.4 or 0.5 merely produced mixed phases comprising spheres and vesicles. However, a pure vesicle morphology was observed when employing **PEG_113_**-rich PISA formulations (see Fig. 2). Furthermore, using **PEG_113_** mole fractions of either 0.6 or 0.7 clearly afforded the smallest vesicles with the narrowest size distributions (lowest DLS polydispersities). However, in our experience, the polydispersity reported by DLS is a rather crude measure of the breadth of a size distribution. For example, the relatively low DLS polydispersities (<0.10) obtained for [*x***PEG_113_** + (1 – *x*) **PEG_45_**] – **PHPMA_400_** vesicles when *x* = 0.7 or 0.8 (Fig. S3†) are clearly inconsistent with the corresponding TEM images (Fig. S4†) since the latter suggest a rather broad size range. Hence we followed the strategy adopted by Gonzato and co-workers,^73^ who utilized small-angle X-ray scattering (SAXS) to compare vesicle size distributions. Accordingly, SAXS patterns were recorded for each of the aqueous PISA formulations reported in Fig. 1 and 2 (see Fig. 4). One striking observation is that the two vesicle dispersions identified by DLS as possessing relatively low polydispersities (*i.e. x* = 0.6 and 0.7) exhibit multiple fringes at intermediate *q*. This is a well-known signature for particles with relatively narrow size distributions since minima arising from the particle form factor are only partially smeared by the particle size distribution. Moreover, the large polydisperse vesicles formed at higher **PEG_113_** mole fractions according to DLS (see Fig. 3) are characterized by SAXS patterns with substantially attenuated minima (see Fig. 4). Thus, these two sizing techniques are in rather good agreement.

While DLS provides information regarding the vesicle size distribution, SAXS provides additional structural information. However, an appropriate scattering model is required to fit SAXS patterns to determine the overall vesicle diameter, size polydispersity, membrane thickness, mean aggregation number, radius of gyration of the stabilizer chains and the solvent volume fraction within the vesicle membrane. TEM analysis (Fig. 2) indicates the presence of two copolymer morphologies: spheres and vesicles. There are well-developed SAXS models for analysing scattering patterns for both types of diblock copolymer nano-objects.^100^,^101^ However, as the current system comprises two types of diblock copolymer chains bearing enthalpically incompatible stabilizer blocks, their spatial distribution within the coronal layer must also be considered. For a vesicle morphology, the **PEG_113_** and **PMPC_28_** stabilizer chains could be either randomly distributed or spatially segregated between the inner and outer vesicle leaflets. Thus, the well-established scattering model for vesicles^101^ requires further refinement. Similarly, adjustment must be made to the scattering model for spheres if this morphology is obtained.^100^

### Small angle X-ray scattering (SAXS) models

In general, the X-ray scattering intensity, *I*(*q*), from a dispersion of uniform nano-objects can be expressed as:1
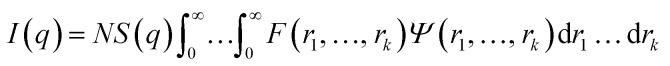
where *F*(*q*, *r*_1_, …, *r*_*k*_) is the form factor, *r*_1_, …, *r*_*k*_ is a set of *k* parameters describing the structural morphology, *Ψ*(*r*_1_, …, *r*_*k*_) is the distribution function, *S*(*q*) is the structure factor and *N* is the number density of nano-objects per unit volume expressed as:2
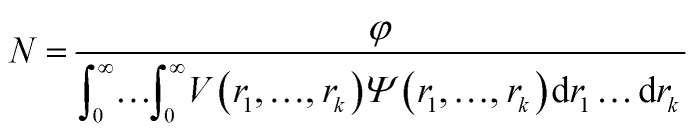
where *V*(*r*_1_, …, *r*_*k*_) is the volume of the nano-objects and *φ* is their volume fraction in the dispersion. The spherical micelle form factor to be used in eqn (1) can be expressed as:^100^,^102^

3 *F*_mic_(*q*, *r*_1_) = *N*_s_^2^*β*_s_^2^*A*_s_^2^(*q*, *r*_1_) + *N*_s_*β*_c_^2^*F*_c_(*q*, *R[combining macron]*_g_) + *N*_s_(*N*_s_ – 1) × *β*_c_^2^*F*_co_(*q*, *r*_1_) + 2*N*_s_^2^*β*_s_*β*_c_*A*_s_(*q*, *r*_1_)*A*_co_(*q*, *r*_1_)*ψ*(*q*, *R*_gblob_)

where *r*_1_ is the core radius of the spherical micelle and *R[combining macron]*_g_ is the averaged radius of gyration of the corona blocks. To model spherical micelles comprising a binary mixture of **PEG_113_** and **PMPC_28_** blocks, the *R[combining macron]*_g_ was calculated based on their relative volume fractions using an approximate radius of gyration for each pure block [*R*_gPEG_ = 2.6 nm and *R*_gPMPC_ = 1.4 nm]. These *R*_g_ values were estimated assuming that the repeat unit length of PMPC is 0.255 nm (the length of two C–C bonds in a *trans* conformation). Thus, the total contour length of a **PMPC_28_** block is *L*_PMPC28_ = 28 × 0.255 nm = 7.15 nm. Similarly, the contour length of an ethylene glycol repeat unit is 0.37 nm (estimated from the known crystal structure of PEG homopolymer),^103^ hence the total contour length of a **PEG_113_** block is *L*_PEG113_ = 113 × 0.37 nm = 41.81 nm. Assuming a mean PMPC Kuhn length of 1.53 nm (based on the known literature value for PMMA)^104^ and a mean PEG Kuhn length of 1.0 nm,^105^ the estimated unperturbed radius of gyration for each block was determined using *R*_g(PEG or PMPC)_ = (contour length × Kuhn length/6)^0.5^. The self-correlation term for the corona blocks is given by the Debye function:4
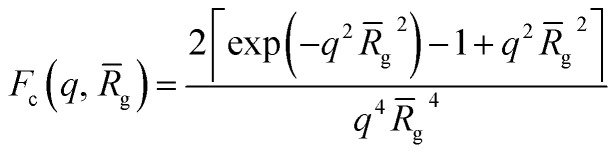



The core and corona block X-ray scattering contrast is given by *β*_s_ = *V*_s_(*ξ*_s_ – *ξ*_sol_) and *β*_c_ = *V[combining macron]*_c_(*ξ̄*_c_ – *ξ*_sol_), respectively. *ξ*_s_, *ξ̄*_c_, and *ξ*_sol_ are the scattering length density (SLD) of the core block (*ξ*_HPMA_ = 11.11 × 10^10^ cm^–2^), the mean SLD of the corona blocks (*ξ*_PMPC_ = 11.6 × 10^10^ cm^–2^ and/or *ξ*_PEG_ = 10.85 × 10^10^ cm^–2^) and the SLD of the solvent (*ξ*_water_ = 9.42 × 10^10^ cm^–2^), respectively. The mean SLD of the corona formed when using binary mixtures of **PEG_113_** and **PMPC_28_** stabilizer blocks was calculated based on the relative volume fractions of these two components. *V*_s_ is the volume of the core block and *V[combining macron]*_c_ is the mean volume of the corona block calculated from the **PEG_113_** and **PMPC_28_** block volumes (*V*_PEG_ and *V*_PMPC_, respectively) using their relative volume fractions. Block copolymer volumes were obtained from the relation 
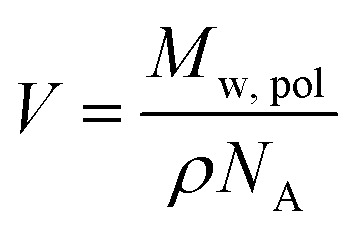
 using the following densities: *ρ*_PMPC_ = 1.28 g cm^–3^, *ρ*_PEG_ = 1.17 g cm^–3^ and *ρ*_HPMA_ = 1.21 g cm^–3^ (see ESI for further details†). Here *M*_w,pol_ is the mean molecular weight of the stabilizer block (**PEG_113_** or **PMPC_28_**) as determined by ^1^H NMR spectroscopy and *N*_A_ is Avogadro's constant. The amplitude of the sphere form factor is used for that of the core self-term:5
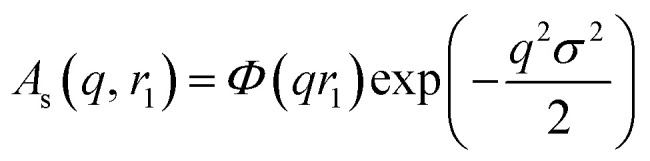
where6
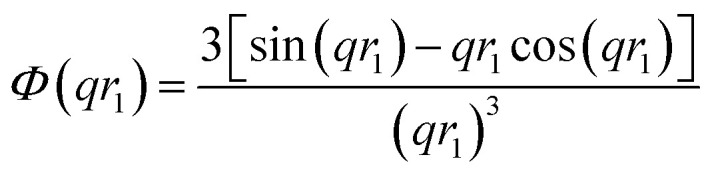



A sigmoidal interface between the two blocks was assumed for the spherical micelle form factor [eqn (3)]. This is described by the exponent term in eqn (5) with a width, *σ*, to account for the decaying scattering length density at the core–shell interface. The *σ* value was fixed at 2.5 Å during fitting. The form factor amplitude for the spherical micelle corona is given by:7
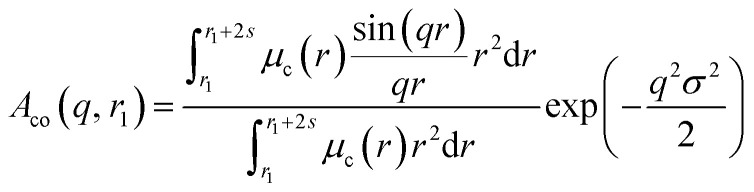
Here *μ*_c_(*r*) denotes the radial profile, which can be expressed by the linear combination of two cubic splines using two fitting parameters *s* and *a* that correspond to the width of the profile and the weight coefficient, respectively. This information, along with the approximate integrated form of eqn (7) can be found elsewhere.^106^,^107^ In principle, randomly distributed **PEG_113_** and **PMPC_28_** stabilizers within the vesicle corona should produce domains with differing SLDs. Such SLD fluctuations within the self-assembled nano-objects would lead to additional scattering at high *q*. Similar structural formation has been incorporated into a well-known scattering model for spherical micelles using a ‘blob’ model.^102^ In this case, it is assumed that the form factor for the fluctuations (‘blobs’) can be described by the known analytical expression for polymer chains. Thus, SLD fluctuations within the micelle corona are incorporated within the spherical micelle form factor [eqn (3)] with the scattering amplitude of the ‘blobs’ expressed as:8
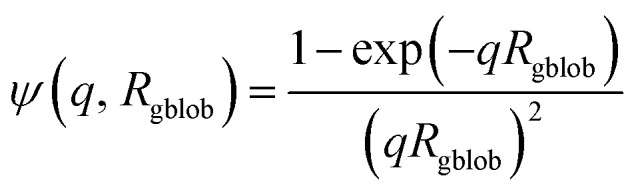
where *R*_gblob_ is the ‘blob’ radius of gyration and the corona form factor is expressed as:

9 *F*_co_(*q*, *r*_1_) = [*n*_blob_(*n*_blob_ – 1)*ψ*^2^(*q*, *R*_gblob_)*A*_co_^2^(*q*, *r*_1_) + *n*_blob_*F*_c_(*q*, *R*_gblob_)]/*n*_blob_^2^

where 
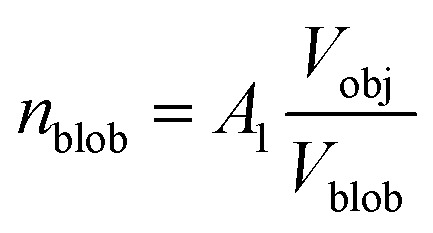
, *A*_1_ is a fitting parameter related to the number of ‘blobs’ formed in the studied nano-object, the ‘blob’ volume is given by 
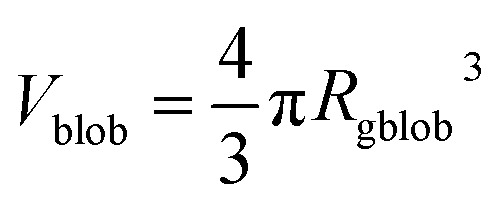
 and *V*_obj_ is equal to the spherical micelle corona volume 
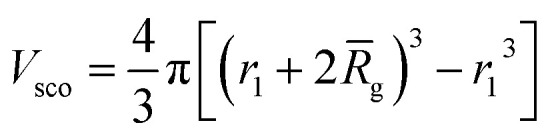
. *F*_c_(*q*, *R*_gblob_) is expressed using a function analogous to that given in eqn (4). The mean aggregation number for the spherical micelles is given by:10
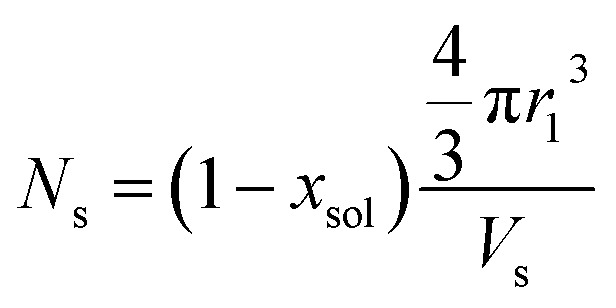
where *x*_sol_ is the volume fraction of solvent (water) in the PHPMA micelle cores. The micelle core radius, *r*_1_, is the only parameter that is assumed to be polydisperse and is described by a Gaussian distribution. Therefore, the polydispersity function in eqn (1) can be written as:11
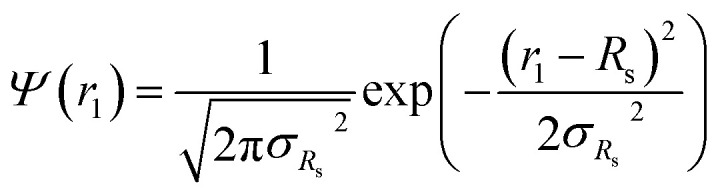
where *R*_s_ is the mean micelle core radius and σ_*R*_s__ is its standard deviation. In accordance with eqn (2), the number density per unit volume for the micelle model is expressed as:12
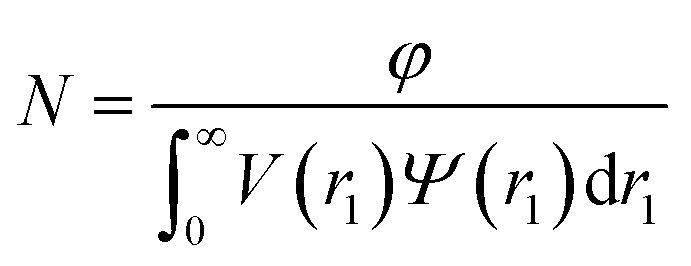
Here *φ* represents the total volume fraction of copolymer forming the spherical micelles and *V*(*r*_1_) is the total volume of copolymer in a spherical micelle:

13 *V*(*r*_1_) = (*V*_s_ + *V[combining macron]*_c_)*N*_s_(*r*_1_)

A structure factor, *S*(*q*), was included in this model to account for the repulsive interactions arising from the anionic carboxylate end-group on each **PMPC_28_** stabilizer chain. Since the micelles are not perfectly centrosymmetric, eqn (1) should be rewritten as:^108^14
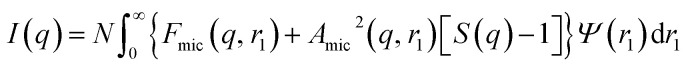
where the scattering amplitude of the spherical micelles is expressed as:

15 *A*_mic_(*q*, *r*_1_) = *N*_s_*β*_s_*A*_s_(*q*, *r*_1_) + *N*_s_*β*_c_*A*_co_(*q*, *r*_1_)*ψ*(*q*, *R*_gblob_)

A hard-sphere structure factor, *S*(*q*) = *S*_PY_(*q*, *R*_PY_, *f*_PY_), (solved using the Percus–Yevick closure relation) was introduced to account for interactions between spherical micelles,^108^ where *R*_PY_ is the effective interparticle correlation radius and *f*_PY_ is the effective volume fraction. Although this structure factor is not strictly correct in this case, it nevertheless provides a useful analytical expression.^109^

For vesicles, the form factor in eqn (1) is given as

16 *F*_v_(*q*, *r*_1_, *r*_2_) = *N*_v_^2^*β*_s_^2^*A*_m_^2^(*q*, *r*_1_, *r*_2_) + *N*_v_(*N*_v_ – 1)*F*_vc_(*q*, *r*_1_, *r*_2_) + 2*N*_v_^2^*β*_s_*A*_m_(*q*, *r*_1_, *r*_2_)*A*_vc_(*q*, *r*_1_, *r*_2_)*ψ*(*q*, *R*_gblob_) + *N*_v_[*f*_PEG_*β*_PEG_^2^*F*_c_(*q*, *R*_gPEG_) + *f*_PMPC_*β*_PMPC_^2^*F*_c_(*q*, *R*_gPMPC_)]

where some terms are the same as those in the spherical micelle model and *F*_c_(*q*, *R*_gPEG_) and *F*_c_(*q*, *R*_gPMPC_) are each expressed using functions that are analogous to eqn (4). Following the original vesicle model,^101^ it is assumed that an equal number of stabilizer chains occupy the outer and the inner vesicle corona. However, the vesicle form factor equation was modified to account for the two different stabilizer blocks (**PEG_113_** and **PMPC_28_**). The amplitude of the membrane self-term is given by:17
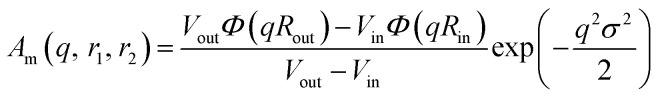
Here 
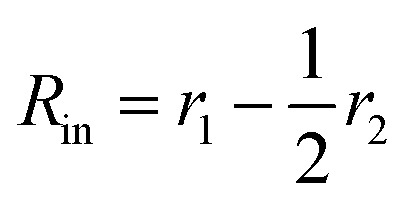
 is the inner radius of the membrane, 
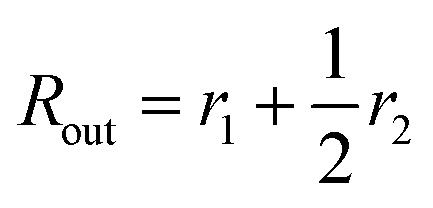
 is the outer radius of the membrane (in this case, *r*_1_ is the radius from the centre of the vesicle to the middle of its membrane and *r*_2_ is the thickness of the hydrophobic part of the membrane), 
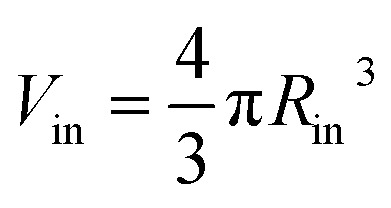
, and 
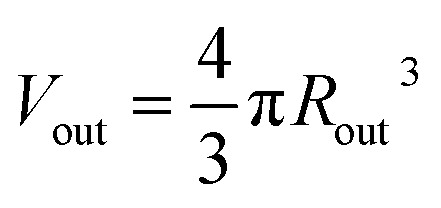
. *Φ*(*qR*_out_) and *Φ*(*qR*_in_) are defined by expressions that are analogous to those used in eqn (6). The mean vesicle aggregation number, *N*_v_(*r*_1_, *r*_2_), is given by:18
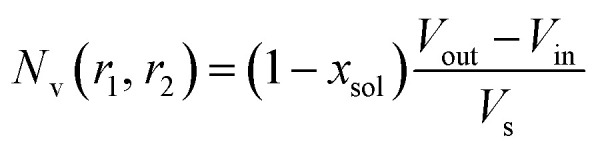
where *x*_sol_ is the volume fraction of solvent (water) within the vesicle membrane. Assuming that there is no penetration of the hydrophilic coronal blocks into the hydrophobic membrane, the amplitude of the vesicle corona self-term is expressed as:19
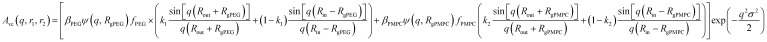
Here *ψ*(*q*, *R*_gPEG_) and *ψ*(*q*, *R*_gPMPC_) are the form factor amplitudes for the PEG and PMPC corona blocks, respectively, expressed using a function that is analogous to that employed in eqn (8). The X-ray scattering contrast for the **PEG_113_** or **PMPC_28_** stabilizer chains is given by *β*_PEG_ = *V*_PEG_(*ξ*_PEG_ – *ξ*_sol_) or *β*_PMPC_ = *V*_PMPC_(*ξ*_PMPC_ – *ξ*_sol_), respectively. In this model, the mole fractions of **PEG_113_**, *x*, and **PMPC_28_**, (1 – *x*), are expressed as *f*_PEG_ and *f*_PMPC_ = 1 – *f*_PEG_, respectively. If *f*_PEG_ ≥ 0.5 the coefficients in eqn (19) indicating the proportion of **PEG_113_** and **PMPC_28_** blocks located within the outer leaflet of the vesicle membrane are expressed as 
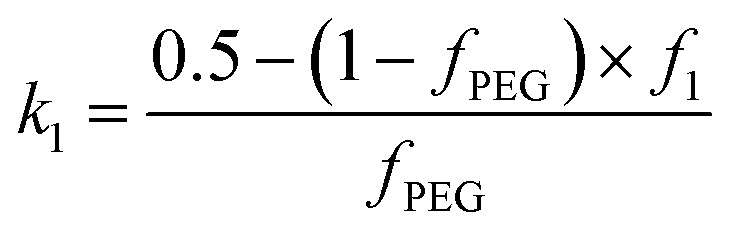
 and *k*_2_ = *f*_1_, respectively. *f*_1_ is a fitting parameter such that 0 ≤ *f*_1_ ≤ 1, where *f*_1_ = 0.5 corresponds to a random distribution of the **PEG_113_** and **PMPC_28_** stabilizer chains between the outer and the inner vesicle leaflets. Importantly, an *f*_1_ value of zero indicates that all **PMPC_28_** stabilizer chains are preferentially located in the inner vesicle leaflet, *i.e.* within the vesicle lumen. In contrast, an *f*_1_ value of unity indicates that these stabilizer chains are exclusively located in the outer vesicle leaflet (Fig. 4B). The form factor for the inner and outer vesicle corona is described as:

20 *F*_vc_(*q*, *r*_1_, *r*_2_) = [*n*_blob_(*n*_blob_ – 1)*ψ*^2^(*q*, *R*_gblob_) × *A*_vc_^2^(*q*, *r*_1_, *r*_2_) + *n*_blob_*F*_c_(*q*, *R*_gblob_)]/*n*_blob_^2^

For such *n*_blob_ calculations, *V*_obj_ is equal to the vesicle corona volume 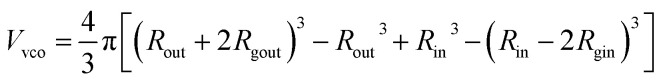. Herein, the mean thickness of the outer and inner vesicle corona were calculated as the average diameter of the outer and inner corona block, 2*R*_gout_ = 2(*f*_PEG_ × *k*_1_ × *R*_gPEG_^3^ + *f*_PMPC_ × *k*_2_ × *R*_gPMPC_^3^)^1/3^ and 2*R*_gin_ = 2[*f*_PEG_(1 – *k*_1_) × *R*_gPEG_^3^ + *f*_PMPC_(1 – *k*_2_) × *R*_gPMPC_^3^]^1/3^, respectively.

For the vesicle model, it was assumed that *R*_v_ (the mean radius from the centre of the vesicle to the middle of the membrane) and *T*_m_ (the mean vesicle membrane thickness) have finite polydispersity. Assuming that each parameter has a Gaussian distribution, the polydispersity function in eqn (1) can be expressed as:21
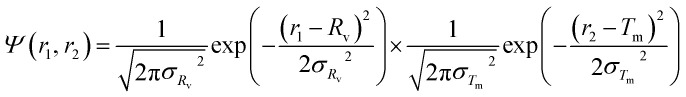
where *σ*_*R*_v__ and *σ*_*T*_m__ are standard deviations. Following eqn (2), the number density per unit volume for the vesicle model is expressed as:22
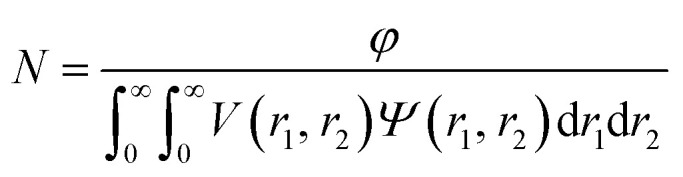
Here *φ* is the total copolymer volume fraction forming the vesicles and *V*(*r*_1_, *r*_2_) is the total volume of copolymer chains within a vesicle:

23 *V*(*r*_1_, *r*_2_) = (*V*_s_ + *f*_PEG_*V*_PEG_ + *f*_PMPC_*V*_PMPC_)*N*_v_(*r*_1_, *r*_2_)

Since the vesicles are significantly larger than spherical micelles, the structure factor for vesicle interactions only makes a significant contribution at low *q*. Unfortunately, this region was not well-resolved in our synchrotron SAXS experiments. Thus, it was assumed for SAXS analysis of the vesicles that the structure factor is close to unity [*S*(*q*) = 1 in eqn (1)]. The programming tools available within Irena SAS macros for Igor Pro were used to implement the scattering models.^110^ Model fittings were performed using the least-squares method.

### SAXS analysis

The above structural models for spheres and vesicles produced reasonably good fits to the corresponding experimental SAXS patterns (Fig. 4). Unfortunately, the first minimum in the form factor at *q* < 0.004 Å^–1^ corresponding to the overall vesicle diameter is not sufficiently resolved to observe the associated secondary minimum at *q* ∼ 0.008 Å^–1^ (Fig. 4). This is likely to be the result of smearing at low *q* (close to the beamstop) caused by the finite X-ray beam cross-section and the pixel size of the X-ray detector. This technical problem has some repercussions for the data fits. To overcome this problem, the most affected data points close to the beamstop (including the first minimum and below) were excluded from the fitting range. The resulting structural parameters were then used to recalculate scattering patterns over the entire experimental *q* range (Fig. 4). A summary of the structural parameters derived from data fits to the SAXS patterns shown in Fig. 4 is provided in Table 1.

**Table 1:** TEM morphology assignment, DLS diameter (*D*_z_) and polydispersity index (PDI) and various structural parameters determined from SAXS analysis of a series of [*x***PEG_113_** + (1 – *x*) **PMPC_28_**] – **PHPMA_400_** nano-objects with PEG mole fractions ranging from 0.0–1.0^*a*^

**PEG_113_** (*x*)	TEM	DLS		SAXS							
	Morphology assignment	*D*_z_/nm	PDI	*R*_s_ ± *σ*_*R*_s__/nm	*R*_v_ ± *σ*_*R*_v__/nm	*σ*_*R*_v__/*R*_v_/%	*T*_m_ ± *σ*_*T*_m__/nm	*σ*_*T*_m__/*T*_m_/%	*D*_s_ or *D*_v_/nm	*R*_gblob_/nm	*f*_1_
0	Spheres	82	0.02	34.0 ± 3.0	—	—	—	—	73.3	—	—
0.1	Spheres	69	0.01	27.9 ± 2.9	—	—	—	—	61.6	4.2	—
0.2	Spheres	69	0.03	28.2 ± 3.1	—	—	—	—	62.6	3.9	—
0.3	Spheres	71	0.03	28.4 ± 3.3	—	—	—	—	63.4	4.1	—
0.4	Spheres & vesicles	92	0.07	n.d.	n.d.	n.d	n.d.	n.d.	n.d.	n.d.	n.d.
0.5	Spheres & vesicles	146	0.08	—	60.9 ± 18.8	31	23.9 ± 3.6	15	156	4.2	0.5*
					61.5 ± 18.8	31	23.7 ± 3.6	15	157		0.14
0.6	Vesicles	189	0.08	—	79.6 ± 10.6	13	22.3 ± 2.8	13	192	2.8	0.5*
					80.1 ± 10.6	13	22.1 ± 2.8	13	193		∼0
0.7	Vesicles	200	0.07	—	81.7 ± 12.6	15	22.1 ± 2.7	12	196	2.9	0.5*
					82.1 ± 12.6	15	21.9 ± 2.7	12	197		∼0
0.8	Vesicles	228	0.13	—	81.8 ± 13.1	16	21.5 ± 2.6	12	196	3.3	0.5*
					82.1 ± 13.1	16	21.5 ± 2.6	12	196		∼0
0.9	Vesicles	331	0.17	—	98.5 ± 35.1	36	21.0 ± 2.4	11	228	3.0	0.5*
					98.7 ± 35.1	36	20.9 ± 2.4	12	229		∼0
1.0	Vesicles	462	0.18	—	185 ± 54	29	20.4 ± 2.8	14	402	—	—

^*a*^ *R*_s_ is the spherical micelle core radius, *R*_v_ is the distance from the centre of the vesicle to the middle of the vesicle membrane and *σ*_R_ denotes the corresponding standard deviations for these two parameters. *T*_m_ is the vesicle membrane thickness and *σ*_*T*_m__ denotes the standard deviation of this parameter. *D*_s_ or *D*_v_ are the sphere or vesicle diameter respectively. *R*_gblob_ is the radius of gyration of the inhomogeneous ‘blobs’ within the coronal layer of stabilizer chains. *f*_1_ is the segregation parameter which indicates the fraction of PMPC stabilizer chains located within the outer corona, and an asterisk (*) indicates when *f*_1_ was fixed at 0.50 during fitting (N.B. ‘n.d.’ denotes ‘not determined’).

The initial morphology assignment by TEM informed our choice of scattering model, with satisfactory data fits being achieved in all but one case (*x* = 0.40). Data fits to the experimental SAXS patterns of the kinetically-trapped spheres obtained using a relatively high proportion of **PMPC_28_** stabilizer chains could be achieved using the blob-modified spherical micelle model (Fig. 4A). Exceptionally, the scattering pattern recorded for the **PMPC_28_**–**PHPMA_400_** spheres (which would not be expected to exhibit SLD variations across the sphere corona) was fitted using the classic spherical micelle model.^100^ For spheres obtained when using a **PEG_113_** mole fraction (*x*) of 0.10–0.30, the structural information derived from such data fits was consistent with DLS studies, suggesting that all three formulations produced spheres with comparable micelle core radii and overall diameters [calculated using *D*_s_ = 2(*R*_s_ + 2*R[combining macron]*_g_)] with relatively low polydispersities of 10–12%. Furthermore, *R*_gblob_ values were comparable to the radii of gyration of the stabilizer chains, indicating that similar inhomogeneous blobs were generated across the sphere coronas.

When fitting vesicles comprising a binary mixture of **PEG_113_** and **PMPC_28_** stabilizer chains, two scenarios were considered when assessing the spatial distribution of these stabilizer chains across the inner and outer vesicle leaflets. Initially, SAXS patterns were fitted assuming a statistical distribution (*f*_1_ = 0.5) of **PEG_113_** and **PMPC_28_** stabilizer chains across the inner and outer leaflets (see Fig. 4C and the upper row of each **PEG_113_** mol fraction between 0.5 and 0.9 in Table 1 for fitting results). Owing to the large number of parameters, *R*_gPEG_ and *R*_gPMPC_ were fixed at their estimated values of 2.6 nm and 1.4 nm, respectively. The second scenario involved fitting the segregation parameter, *f*_1_, using the data fit obtained in the first scenario as a starting point (see Fig. 4D and the lower row of each **PEG_113_** mole fraction between 0.5 and 0.9 in Table 1 for fitting results). This conservative approach was adopted owing to the large number of parameters involved in the refined vesicle model. In addition, *R*_gblob_ and its related fitting parameter were fixed when fitting *f*_1_.

Clearly, the data fits achieved for both the first and second scenarios appear to be very similar (compare Fig. 4C and D). However, it is emphasized that the minimized chi-squared value was always reduced by about 5% in the latter case, with the vesicle radius (*R*_v_), membrane thickness (*T*_m_) and their associated polydispersities (*σ*_*R*_v__ and *σ*_*T*_m__) varying very little when fitting *f*_1_ (Table 1). Given that the main contribution to the overall X-ray scattering comes from the hydrophobic PHPMA chains within the vesicle membranes rather than the hydrophilic **PEG_113_** and **PMPC_28_** stabilizer chains, this apparently modest reduction in the chi-squared value is considered to be significant. Thus, fitting *f*_1_ simply provides a statistically better fit, rather than revealing any new structural features arising from the microphase separation of the **PEG_113_** and **PMPC_28_** stabilizer chains across the vesicle membrane. Typically, *f*_1_ tended towards zero whenever this parameter was not constrained. According to Fig. 4B and Table 1, these very low *f*_1_ values determined for *x* = 0.6–0.9 indicate that the majority of the **PMPC_28_** chains are located within the vesicle inner leaflet, suggesting substantial enthalpic incompatibility between the **PEG_113_** and **PMPC_28_** blocks. In the case of the (0.5 **PEG_113_** + 0.5 **PMPC_28_**) – **PHPMA_400_** formulation (*i.e. x* = 0.5 in Table 1, for which *f*_1_ = 0.14), a plausible explanation is that there are more **PMPC_28_** chains than can be accommodated within the inner leaflet, causing some of these chains to occupy the outer leaflet despite the presence of the **PEG_113_** chains. Alternatively, TEM analysis indicated that a minor population of spheres are also present in this case (Fig. 2), which might be expected to affect the *f*_1_ value [N.B. A satisfactory data fit could nevertheless be obtained using the modified vesicle model simply because the much smaller spheres make a negligible contribution to the X-ray scattering].

According to TEM analysis, the first pure vesicle phase is formed by the (0.6 **PEG_113_** + 0.4 **PMPC_28_**) – **PHPMA_400_** formulation. Interestingly, these vesicles also exhibit the lowest standard deviation (*σ*_*R*_v__) for the mean vesicle radius (*R*_v_). In this case, the size polydispersity (which is calculated as a variance, *i.e. σ*_*R*_v__/*R*_v_ × 100%) is 13%. SAXS analysis indicates higher polydispersities as the **PEG_113_** mole fraction is increased up to 0.9. Moreover, the *f*_1_ data fits suggest that optimum microphase separation is achieved for the (0.6 **PEG_113_** + 0.4 **PMPC_28_**) – **PHPMA_400_** formulation, which corresponds to the formation of vesicles with the lowest polydispersity. Thus, the above suggestion that the *x* = 0.5 formulation simply contains too many **PMPC_28_** stabilizer chains to be accommodated within the inner leaflet of the vesicles seems to be physically reasonable. Conversely, the *x* = 0.7 formulation contains too few **PMPC_28_** chains to fully occupy the inner leaflet, thus requiring some **PEG_113_** chains to be co-located within the vesicle lumen. As the **PEG_113_** mole fraction is increased, the **PEG_113_** and **PMPC_28_** chains are increasingly unable to maintain complete microphase separation across the membrane, despite the majority of the **PMPC_28_** chains being located within the vesicle lumen. This suggests that the enthalpic incompatibility between the **PEG_113_** and **PMPC_28_** chains drives the formation of relatively small, low polydispersity vesicles.

The fitted *R*_gblob_ values for vesicles comprising both **PEG_113_** and **PMPC_28_** stabilizer blocks were comparable when assuming *f*_1_ = 0.5. These values are also similar to those determined for the spheres, indicating that similar inhomogeneous ‘blobs’ are produced within both types of coronal layers. In addition to confirming the formation of larger, more polydisperse vesicles when using higher **PEG_113_** mole fractions (*x* > 0.7), SAXS analysis also indicated a monotonic (albeit modest) reduction in vesicle membrane thickness with increasing **PEG_113_** mole fraction. The overall vesicle diameter was calculated using *D*_v_ = 2(*R*_v_ + 0.5*T*_m_ + 2*R*_gPEG_). *R*_gPEG_ was used as the *f*_1_ data fits suggest that, in most cases, the vesicle outer leaflet contains solely **PEG_113_** chains. Allowing for the effect of polydispersity, these SAXS-derived volume-average diameters are in reasonably good agreement with the *z*-average diameters reported by DLS (Table 1). As expected, the biggest deviations are observed for relatively large polydisperse vesicles, because DLS is more biased towards larger nano-objects.

### Effect of pH and salt on colloidal stability of vesicles

**Fig. 5:**
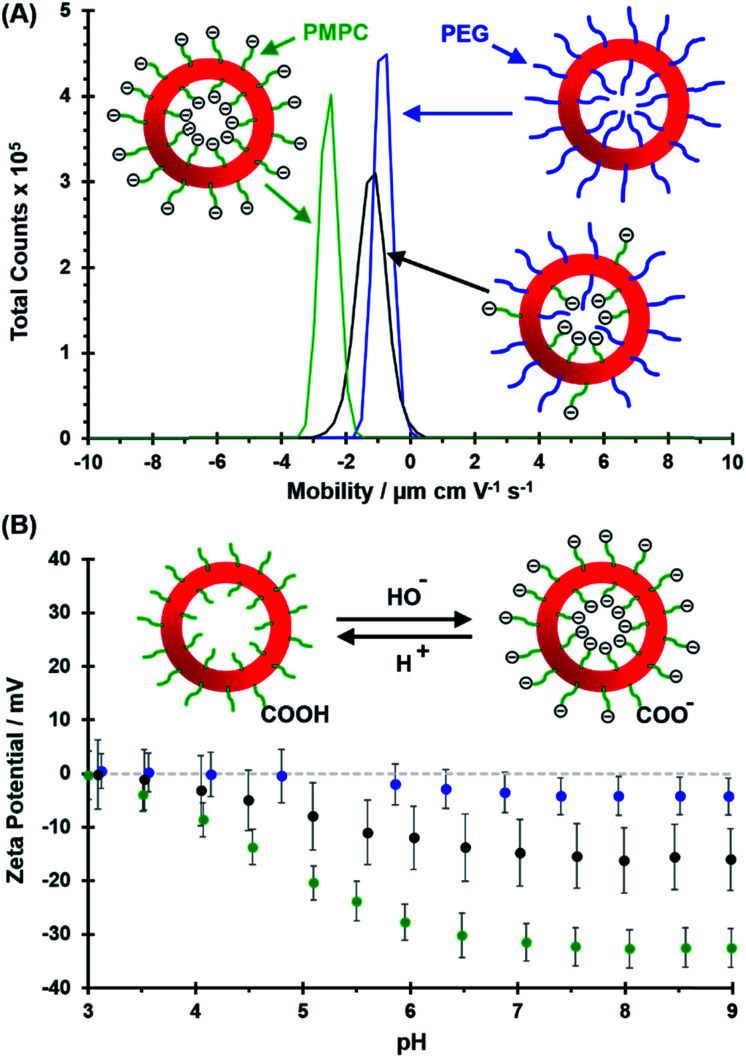
(A) Electrophoretic mobility distributions (determined at pH 7.0) and (B) corresponding zeta potential *vs.* pH curves obtained for **PEG_113_**–**PHPMA_400_** vesicles (blue distribution), [0.6 **PEG_113_** + 0.4 **PMPC_28_**] – **PHPMA_400_** vesicles (black distribution) and **PMPC_28_**–**PHPMA_450_** vesicles (green distribution). Measurements were conducted on 0.1% w/w aqueous dispersions prepared by dilution using an aqueous solution of 1 mM KCl. In (A), ionization of the COOH end-group on each PMPC chain at pH 7.0 is indicated by the terminal negative charge. In the inset cartoon shown in (B), just one COOH (or anionic carboxylate) group per vesicle is shown for clarity.

Electrophoretic mobility distributions (determined at pH 7.0) are shown for the **PEG_113_**–**PHPMA_400_** vesicles, [0.6 **PEG_113_** + 0.4 **PMPC_28_**] – **PHPMA_400_** vesicles and **PMPC_28_**–**PHPMA_450_** vesicles in Fig. 5A. The former vesicles have a relatively low mobility that lies close to zero (see blue distribution). In contrast, the latter vesicles exhibit a distinctly negative mobility owing to ionization of the terminal carboxylic acid group at pH 7.0 (see green distribution). Finally, the [0.6 **PEG_113_** + 0.4 **PMPC_28_**] – **PHPMA_400_** vesicles exhibit intermediate behavior, with a mobility closer to that of the **PEG_113_**–**PHPMA_400_** vesicles (see red distribution). It is perhaps also worth emphasizing that the unimodal nature of this latter distribution is consistent with entropic mixing of the **PEG_113_**–**PHPMA_400_** and **PMPC_28_**–**PHPMA_400_** chains to form hybrid vesicles, rather than the formation of two distinct populations of **PEG_113_**–**PHPMA_400_** and **PMPC_28_**–**PHPMA_400_** nano-objects. Similar observations were reported by Semsarilar and co-workers when preparing hybrid vesicles using binary mixtures of polyelectrolytic and non-ionic steric stabilizers.^111^,^112^

The corresponding zeta potential *vs.* pH curves determined for each type of vesicle are shown in Fig. 5B. These three curves are consistent with the mobility data. Thus, the **PEG_113_**–**PHPMA_400_** vesicles exhibit zeta potentials close to zero, as expected for the non-ionic PEG chains. In contrast, the **PMPC_28_**–**PHPMA_450_** vesicles exhibit quite strongly negative zeta potentials (*e.g.* –33 mV at pH 9) owing to ionization of the carboxylic acid end-group (p*K*_a_ ∼ 4.7)^92^ on the PMPC chains. Finally, the hybrid [0.6 **PEG_113_** + 0.4 **PMPC_28_**] – **PHPMA_400_** vesicles exhibit only weakly negative zeta potentials (–16 mV at pH 9). This is consistent with most of the PMPC chains being preferentially located within the lumen, rather than being expressed at the outer leaflet of such vesicles.

**Fig. 6:**
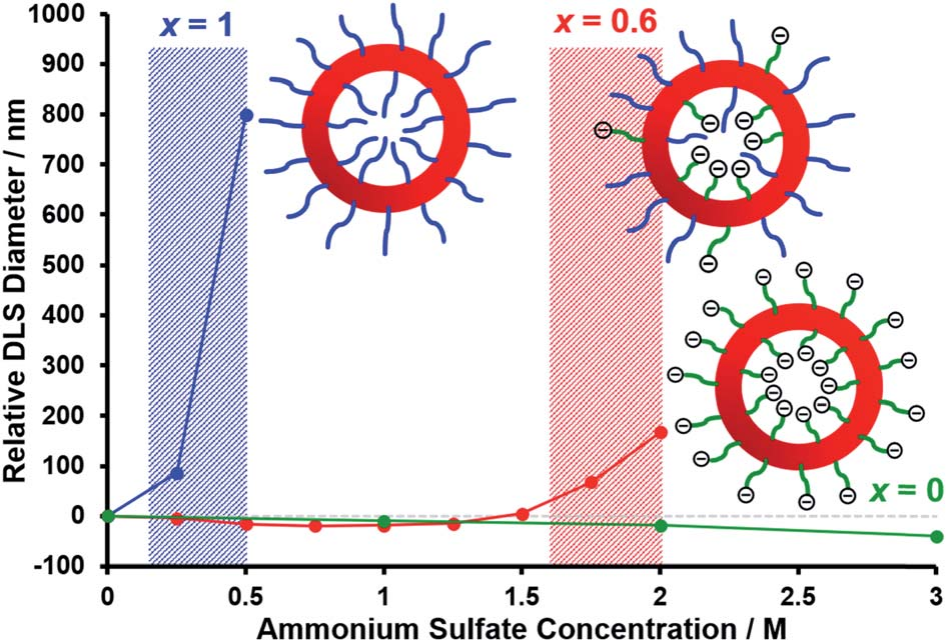
Relative change in intensity-average diameter with added salt normalized to that determined in deionized water for **PEG_113_**–**PHPMA_400_** vesicles (blue data set), [0.6 **PEG_113_** + 0.4 **PMPC_28_**] – **PHPMA_400_** vesicles (red data set), and **PMPC_28_**–**PHPMA_450_** vesicles (green data set). The shaded areas indicate the onset of vesicle flocculation. DLS measurements were conducted on 0.1% w/w aqueous dispersions containing 0 to 3.0 M ammonium sulfate.

Fig. 6 shows the relative change in intensity-average diameter with added salt (up to 3.0 M ammonium sulfate) for **PEG_113_**–**PHPMA_400_** vesicles (blue curve), [0.6 **PEG_113_** + 0.4 **PMPC_28_**] – **PHPMA_400_** vesicles (red curve), and **PMPC_28_**–**PHPMA_450_** vesicles (green curve). All data are normalized to the intensity-average diameter of each type of vesicle as determined in deionized water. It is well known that PEG can be readily salted out in the presence of sulfate anions,^113^ whereas PMPC is highly tolerant to added salt up to 5 M.^114^,^115^ Thus the addition of just 0.50 M ammonium sulfate leads to a substantial increase in the apparent size of the **PEG_113_**–**PHPMA_400_** vesicles, indicating significant flocculation. In contrast, the **PMPC_28_**–**PHPMA_450_** vesicles remain colloidally stable even in the presence of 3.0 M ammonium sulfate. Importantly, the [0.6 **PEG_113_** + 0.4 **PMPC_28_**] – **PHPMA_400_** vesicles undergo incipient flocculation in the presence of 2.0 M ammonium sulfate. This suggests that most of the PEG chains are expressed at the outer leaflet of the vesicle membrane, which is consistent with the aqueous electrophoresis data shown in Fig. 5.

## Conclusions

In summary, judicious use of a binary mixture of a relatively long non-ionic PEG steric stabilizer and a relatively short zwitterionic PMPC steric stabilizer enables the rational synthesis of rather small (<200 nm diameter) hybrid diblock copolymer vesicles with a relatively narrow size distribution (size polydispersity = 13–16%) at 10% w/w solids in aqueous solution *via* polymerization-induced self-assembly. Aqueous electrophoresis and salt-induced flocculation studies provide evidence for the relatively long PEG chains being preferentially expressed at the outer leaflet of the vesicle membrane. SAXS studies confirm that systematic variation of the relative proportions of the zwitterionic and non-ionic steric stabilizers is required to achieve optimal control over the vesicle size distribution. SAXS analysis also provides further evidence for confinement of most of the PMPC chains to the inner leaflet of the vesicles. Importantly, control experiments conducted using a binary mixture of chemically identical long and short PEG stabilizer blocks only produced relatively large vesicles which are less useful for potential biomedical applications. Thus, enthalpic incompatibility between the two types of steric stabilizers appears to offer a decisive advantage in this context. We anticipate that the reproducible and scalable synthesis of highly biocompatible small vesicles with relatively narrow size distributions reported herein will drive new developments in the field of nanobiotechnology.

## Conflicts of interest

There are no conflicts to declare.

## Supplementary Material

Supplementary information
