# Supplementary material for: Enthalpic incompatibility between two steric stabilizer blocks provides control over the vesicle size distribution during polymerization-induced self-assembly in aqueous media
Source: Chem Sci. 2020 Jun 29;11(39):10821–34. doi: 10.1039/d0sc01320j (PMC7654191; doi:10.1039/d0sc01320j)
Supplement: Supplementary file 1 [file SC-011-D0SC01320J-s001.pdf]

Electronic Supplementary Material (ESI) for Chemical Science.
This journal is © The Royal Society of Chemistry 2020

Supporting Information for:

Enthalpic Incompatibility Between Two Steric Stabilizer Blocks Provides Control Over the Vesicle Size Distribution During Polymerization-Induced Self-Assembly in Aqueous Media

D. L. Beattie, O. O. Mykhaylyk and S. P. Armes*

Experimental

Materials

2-(Methacryloyloxy)ethyl phosphorylcholine (MPC) was kindly donated by Biocompatibles Ltd. (Farnham, UK) and was used as received. 4-Cyano-4-(2-phenylethanesulfanyl-thiocarbonyl)sulfanylpentanoic acid (PETTC) RAFT agent was prepared in-house as previously reported.[1] The PEG_{113} macro-CTA was synthesized as previously described.[2] 2-Hydroxypropyl methacrylate (HPMA) monomer was kindly provided by GEO Specialty Chemicals (Hythe, UK). The 2,2'-azobis[2-(2-imidazolin-2-yl)propane] dihydrochloride (VA-044) initiator was purchased from Fluorochem (Glossop, UK). Deionized water was obtained from an Elgastat Option 3A water purification unit and used for all experiments. CD_3OD was purchased from Cambridge Isotope Laboratories (UK). Chloroform, ethanol and methanol were all HPLC-grade and obtained from VWR Chemicals (UK).

Methods

Synthesis of poly(2-(methacryloyloxy)ethyl phosphorylcholine) (PMPC) via RAFT solution polymerization. MPC monomer (6.00 g, 20.3 mmol), PETTC (0.300 g, 0.88 mmol), and ACVA initiator (0.050 g, 0.17 mmol, PETTC/ACVA = 5.0) were dissolved in ethanol (9.524 g) to produce a 40% w/w solution in a 50 mL round-bottomed flask. This flask was sealed, immersed in ice and the reaction mixture was degassed via nitrogen sparge for 30 min before placing the flask in an oil bath set at 75 °C. After continuous stirring at this temperature for 80 min, the polymerization was quenched by exposing the contents of the flask to air while cooling to 20 °C. ^{1}H NMR spectroscopy studies indicated a final MPC conversion of 75%. The crude PMPC was precipitated into a ten-fold excess of acetone before washing three times with a 7:1 acetone/water solution to remove unreacted MPC monomer. Residual acetone was removed under reduced pressure, then the purified PMPC precursor was dissolved in deionized water and freeze-dried overnight to produce a yellow solid. ^{1}H NMR analysis indicated a mean degree of polymerization of 28. GPC analysis (refractive index detector, 3:1 chloroform/methanol eluent) indicated an M_n of 5,100 g mol^{-1} and an M_w/M_n of 1.08.

Synthesis of [*x* PEG_{113} + (1 - *x*) $PMPC_{28}$] – $PHPMA_{400}$ diblock copolymer nanoparticles via RAFT aqueous dispersion polymerization of HPMA. For a typical RAFT aqueous dispersion polymerization of HPMA targeting [0.60 PEG_{113} + 0.40 $PMPC_{28}$] – $PHPMA_{400}$ vesicles at 10 % w/w solids, the following protocol was utilized. PEG_{113} macro-CTA (30.0 mg, 6.0 µmol), $PMPC_{28}$ macro-CTA (32.0 mg, 4.0 µmol), HPMA monomer (536 mg, 3.70 mmol) and VA-044 initiator (1.00 mg, 3.1 µmol, macro-CTA/VA-044 = 3.0) were dissolved in deionized water (5.392 g) in a vial and the solution pH was adjusted to 6.8 using 0.1 M NaOH. This vial was sealed and immersed in ice and the reaction mixture was degassed via nitrogen sparge for 30 min before placing the vial in an oil bath set at 50 °C. After continuous stirring at this temperature for 4 h, the polymerization was quenched by exposing the contents of the vial to air while cooling to 20 °C. ^{1}H NMR spectroscopy studies indicated a final HPMA conversion of more than 99%. A series of [*x* PEG_{113} + (1 - *x*) $PMPC_{28}$] – $PHPMA_{400}$ nanoparticles were prepared by systematically varying the PEG_{113} mol fraction (*x*) between 1 and 0. The [*x* PEG_{113} + (1 - *x*) $PMPC_{28}$] – $PHPMA_{400}$ chains were analyzed by GPC (refractive index detector, 3:1 chloroform/methanol eluent) without further purification (see Table S1) The [*x* PEG_{113} + (1 - *x*) $PMPC_{28}$] – $PHPMA_{400}$ nanoparticles were characterized by DLS, TEM, SAXS and aqueous electrophoresis.

Synthesis of $PMPC_{28}$-$PHPMA_{450}$ diblock copolymer vesicles via RAFT aqueous dispersion polymerization of HPMA. $PMPC_{28}$ macro-CTA (80.0 mg, 9.3 µmol), HPMA monomer (600 mg, 4.2 mmol) and VA-044 initiator (1.0 mg, 3.1 µmol, macro-CTA/VA-044 = 3.0) were dissolved in deionized water (2.0418 g) in a vial to produce a 25 % w/w solution. This vial was sealed and immersed in ice and the reaction mixture was degassed via nitrogen sparge for 30 min before placing the vial in an oil bath set at 50 °C. After continuous stirring at this temperature for 4 h, the polymerization was quenched by exposing the contents of the vial to air while cooling to 20 °C. ^{1}H NMR spectroscopy studies indicated a final HPMA conversion of more than 99%. The $PMPC_{28}$-$PHPMA_{450}$ chains were analyzed by GPC (refractive index detector, 3:1 chloroform/methanol eluent) without further purification (M_n = 43,200 g mol^{-1}; M_w/M_n = 2.47). The $PMPC_{28}$-$PHPMA_{450}$ vesicles were characterized by DLS, aqueous electrophoresis, TEM and SAXS.

Synthesis of [*x* PEG_{113} + (1 - *x*) PEG_{45}] – $PHPMA_{400}$ diblock copolymer nanoparticles via RAFT aqueous dispersion polymerization of HPMA. For a typical RAFT aqueous dispersion polymerization of HPMA targeting [0.80 PEG_{113} + 0.20 PEG_{45}] – $PHPMA_{400}$ vesicles at 10% w/w solids, the following protocol was utilized. PEG_{113} macro-CTA (40.0 mg, 7.4 µmol), PEG_{45} macro-CTA (4.0 mg, 1.9 µmol), HPMA monomer (536 mg, 3.70 mmol) and VA-044 initiator (1.00 mg, 3.1 µmol, macro-CTA/VA-044 = 3.0) were dissolved in deionized water (5.232 g) in a sample vial. This vial was then sealed, immersed in ice and the reaction mixture degassed via nitrogen sparge for 30 min before placing the vial in an oil bath set at 50 °C. After continuous stirring at this temperature for 4 h, the polymerization was quenched by exposing the contents of the vial to air while cooling to 20 °C. ^{1}H NMR spectroscopy studies indicated a final HPMA conversion of more than 99%. A series of [*x* PEG_{113} + (1 - *x*) PEG_{45}] – $PHPMA_{400}$ vesicles were prepared by systematically varying the PEG_{113} mol fraction (*x*) between 1 and 0. GPC analysis (refractive index detector, 3:1 chloroform/methanol eluent) was conducted on

the dissolved copolymer chains without further purification (see Table S2) and the [x PEG_{113} + (1 - x) PEG_{45}] – $PHPMA_{400}$ vesicles were characterized by DLS and TEM.

Characterisation Methods

^{1}H NMR Spectroscopy. ^{1}H NMR spectra were recorded in CD_3OD at 298 K using a 400 MHz Bruker Avance-400 spectrometer (64 scans averaged per spectrum).

Gel Permeation Chromatography (GPC). GPC analysis of the diblock copolymer chains was conducted at 35 °C using a 3:1 chloroform/methanol eluent containing 2 mM LiBr at a flow rate of 1.0 mL min^{-1}. The instrument comprised an Agilent 1260 GPC system, two Agilent PL gel 5 µm MIXED-C columns connected in series with a guard column, a refractive index (RI) detector and a variable wavelength detector set to 298 nm. Calibration was achieved using ten near-monodisperse poly(methyl methacrylate) (PMMA) standards with M_n values ranging from 625 to 618 000 g mol^{-1}.

Dynamic light scattering (DLS). Measurements were performed on 0.10% w/v aqueous dispersions using a Malvern Zetasizer NanoZS instrument equipped with a 4 mW He-Ne laser (wavelength λ = 633 nm) and an avalanche photodiode detector. Measurements were recorded using disposable cuvettes at 25 °C using a fixed scattering angle of 173°. The Stokes-Einstein equation was used to calculate intensity-average hydrodynamic diameters, which were averaged over three consecutive runs comprising ten measurements per run.

Aqueous Electrophoresis. Mobilities were determined using a Malvern Zetasizer NanoZS instrument at 25 °C using 0.10% w/w aqueous dispersions containing 1 mM KCl as background electrolyte. The solution pH was adjusted by addition of HCl or NaOH as required. Zeta potentials were calculated from mobilities using the Henry equation assuming that the Smoluchowski approximation was valid. Data were averaged over three consecutive runs comprising ten measurements per run.

Transmission Electron Microscopy (TEM). Dispersions were diluted to 0.30% w/w using deionized water (pH 6) at 20 °C. Copper-palladium TEM grids were surface-coated with a thin film of carbon in-house and then plasma glow-discharged for 30 s to produce a hydrophilic surface. A 7 µL droplet of a 0.30% w/w aqueous dispersion of vesicles was deposited onto the surface of each grid for 1 min before blotting with filter paper to remove excess liquid. A 7 µL droplet of a 0.75% w/v aqueous uranyl formate solution was then applied as a negative stain for 25 s before careful blotting and drying using a vacuum hose. Imaging was performed using a FEI Tecnai Spirit 2 microscope operating at 80 kV and equipped with an Orius SC1000B camera.

Density Measurements. Density measurements were recorded for both $PMPC_{28}$ and PEG_{113} using a DMA 5000 M liquid density meter (Anton-Paar Ltd., Graz, Austria) calibrated using deionized water. Aqueous stock solutions of these homopolymers of 1-20% w/w concentration were made up for density measurements, allowing extrapolation to the solid-state density at 100%. The density of the PHPMA homopolymer was determined by helium pycnometry using an AccuPyc 1330 instrument (Micrometrics, Norcross, USA).

Small Angle X-ray Scattering (SAXS). SAXS experiments were performed at a national synchrotron facility (station I22, Diamond Light Source, Didcot, Oxfordshire, UK). The scattered monochromatic x-ray radiation (wavelength λ = 0.124 nm, with *q* ranging from 0.015 to 1.2 nm^{-1}, where $q = 4\pi \sin\theta/\lambda$ is the length of the scattering vector and θ is one-half of the scattering angle) was collected using a 2D Pilatus 2M pixel detector (Dectris, Switzerland). Measurements were conducted on 1.0% w/w aqueous copolymer dispersions in 2 mm diameter capillary sample holders. All scattering patterns were reduced and normalized using standard protocols provided by the beamline facility staff, with further analysis being performed using the Irena SAS macro for Igor Pro.

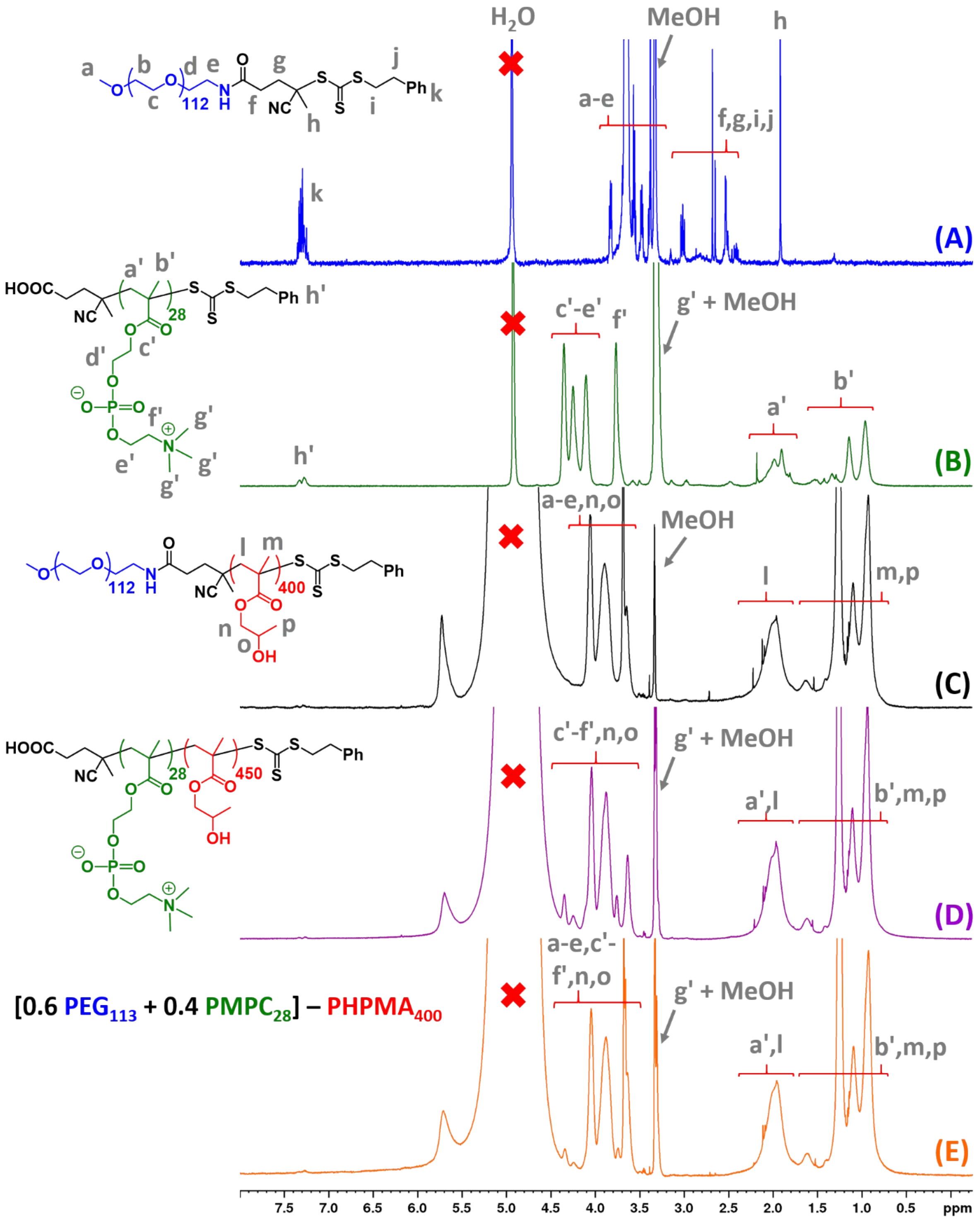

Figure S1. Assigned ^{1}H NMR spectra recorded in d_4-methanol for (A) the PEG_{113} precursor, (B) the $PMPC_{28}$ precursor, (C) a PEG_{113}-$PHPMA_{400}$ diblock copolymer, (D) a $PMPC_{28}$-$PHPMA_{450}$ diblock copolymer and (E) a [0.6 PEG_{113} + 0.4 $PMPC_{28}$] – $PHPMA_{400}$ diblock copolymer.

Table S1. Summary of the 3:1 chloroform/methanol GPC data (refractive index detector; expressed relative to PMMA calibration standards) obtained for PEG_{113} macro-CTA, $PMPC_{28}$ macro-CTA, $PMPC_{28}$-$PHPMA_{450}$ diblock copolymer and a series of ten [x PEG_{113} + (1 – x) $PMPC_{28}$] – $PHPMA_{400}$ diblock copolymers for which x = 0.1 – 1.0. The colors correspond to the various chromatograms shown in Figure S2.

**PISA Formulation**	M_n / g mol^{-1}	M_w / M_n
PEG_{113} precursor	10,600	1.07
$PMPC_{28}$ precursor	5,100	1.08
PEG_{113} – $PHPMA_{400}$	45,400	1.45
(0.9 PEG_{113} + 0.1 $PMPC_{28}$) – $PHPMA_{400}$	48,800	1.37
(0.8 PEG_{113} + 0.2 $PMPC_{28}$) – $PHPMA_{400}$	50,800	1.37
(0.7 PEG_{113} + 0.3 $PMPC_{28}$) – $PHPMA_{400}$	50,400	1.40
(0.6 PEG_{113} + 0.4 $PMPC_{28}$) – $PHPMA_{400}$	52,300	1.41
(0.5 PEG_{113} + 0.5 $PMPC_{28}$) – $PHPMA_{400}$	49,600	1.48
(0.4 PEG_{113} + 0.6 $PMPC_{28}$) – $PHPMA_{400}$	48,500	1.53
(0.3 PEG_{113} + 0.7 $PMPC_{28}$) – $PHPMA_{400}$	48,600	1.56
(0.2 PEG_{113} + 0.8 $PMPC_{28}$) – $PHPMA_{400}$	44,000	1.76
(0.1 PEG_{113} + 0.9 $PMPC_{28}$) – $PHPMA_{400}$	39,900	1.96
$PMPC_{28}$ – $PHPMA_{400}$ (at 10% w/w solids)	38,600	2.08
$PMPC_{28}$ – $PHPMA_{450}$ (at 25% w/w solids)	43,200	2.47

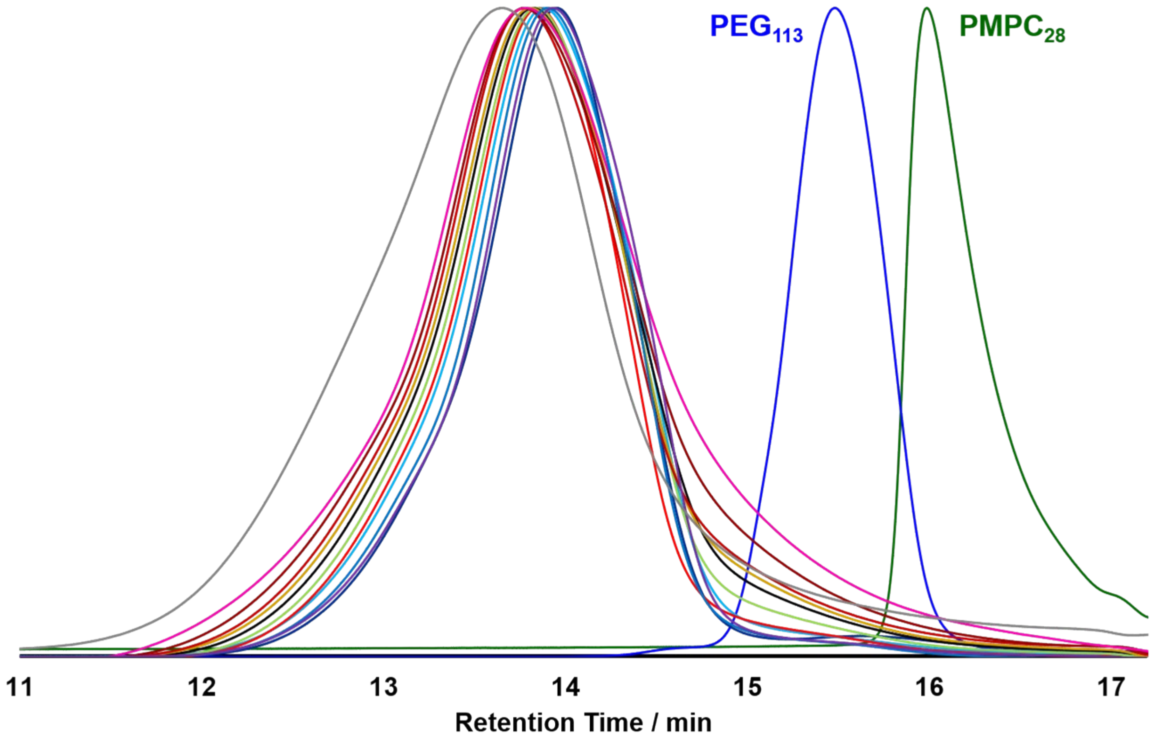

Figure S2. 3:1 Chloroform/methanol GPC curves obtained for the various entries shown in Table S1, including PEG_{113} macro-CTA, $PMPC_{28}$ macro-CTA, $PMPC_{28}$-$PHPMA_{450}$ diblock copolymer and a series of ten [x PEG_{113} + (1 – x) $PMPC_{28}$] – $PHPMA_{400}$ diblock copolymers for which x = 0.1 – 1.0.

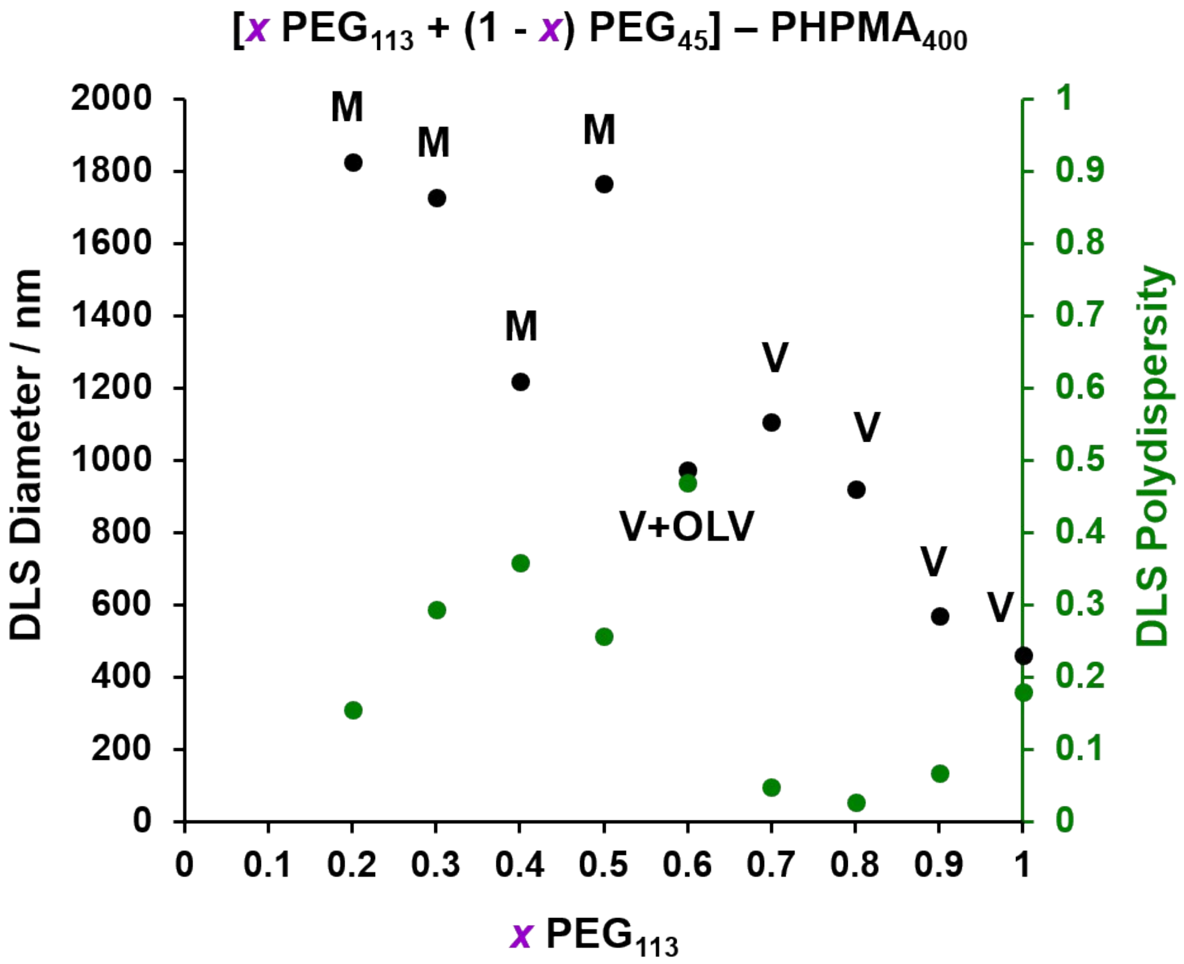

Figure S3. Effect of systematically varying the PEG_{113} mol fraction on the size of the resulting [*x* PEG_{113} + (1 - *x*) PEG_{45}] – $PHPMA_{400}$ diblock copolymer nano-objects as judged by DLS. Intensity-average diameters and polydispersities were determined for 0.1% w/w aqueous dispersions diluted from 10% w/w dispersions using deionized water. **S** indicates spheres, **M** indicates a mixed phase of spheres, worms and large particles, and **V** indicates that vesicles were the predominant morphology. **OLV** denotes the presence of oligolamellar vesicles.

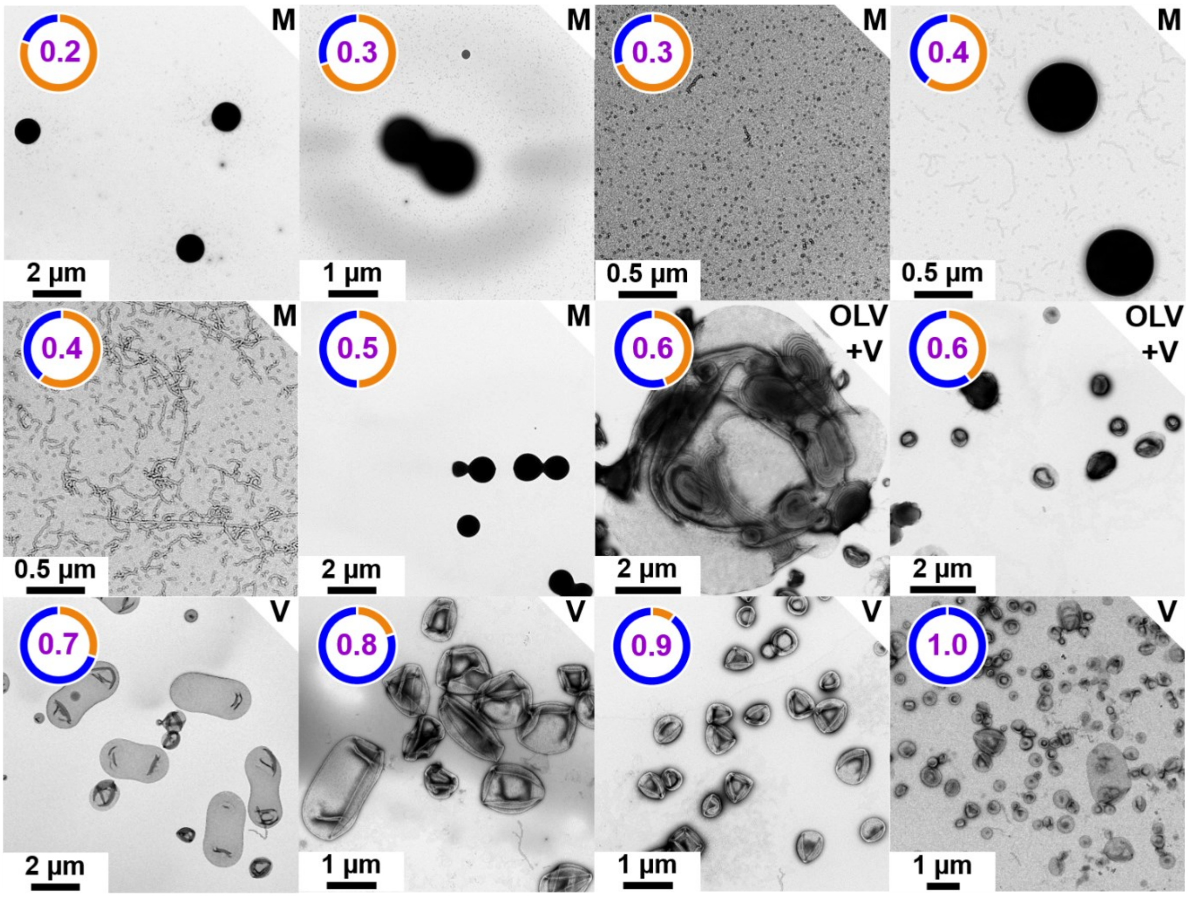

Figure S4. Representative TEM-images recorded for [*x* **PEG_{113}** + (1 - *x*) **PEG_{45}**] – **$PHPMA_{400}$** diblock copolymer nano-objects prepared at 10% w/w solids via RAFT aqueous dispersion polymerization of HPMA at 50 °C while systematically varying the mol fraction (*x*) of the **PEG_{113}** steric stabilizer block from 0.2 to 1.0. The number in purple denotes *x*, while **S** indicates spheres, **M** indicates a mixed phase of spheres, worms and large particles, and **V** indicates that vesicles were the predominant morphology. **OLV** denotes the presence of oligolamellar vesicles. The limitations of using DLS to assess vesicle size distributions become clear when inspecting the TEM images obtained for *x* = 0.7 and 0.8. In both cases, DLS polydispersities of less than 0.10 were obtained (see Table 1) yet rather polydisperse, non-spherical (tube-like) vesicular structures are observed. For this reason, we prefer to use SAXS to assess the breadth of vesicle size distributions (see main manuscript).

Table S2. Summary of the 3:1 chloroform/methanol GPC data (refractive index detector; expressed relative to PMMA calibration standards) obtained for PEG_{113} macro-CTA, PEG_{45} macro-CTA, and a series of nine [x PEG_{113} + (1 – x) PEG_{45}] – $PHPMA_{400}$ diblock copolymers for which x = 0.2 – 1.0.

PISA Formulation	M_n / g mol^{-1}	M_w/M_n
PEG_{113} precursor	10,600	1.07
PEG_{45} precursor	5,000	1.10
PEG_{113} – $PHPMA_{400}$	45,400	1.45
(0.9 PEG_{113} + 0.1 PEG_{45}) – $PHPMA_{400}$	49,100	1.35
(0.8 PEG_{113} + 0.2 PEG_{45}) – $PHPMA_{400}$	46,700	1.37
(0.7 PEG_{113} + 0.3 PEG_{45}) – $PHPMA_{400}$	51,900	1.34
(0.6 PEG_{113} + 0.4 PEG_{45}) – $PHPMA_{400}$	48,800	1.43
(0.5 PEG_{113} + 0.5 PEG_{45}) – $PHPMA_{400}$	44,000	2.24
(0.4 PEG_{113} + 0.6 PEG_{45}) – $PHPMA_{400}$	45,800	2.28
(0.3 PEG_{113} + 0.7 PEG_{45}) – $PHPMA_{400}$	47,300	2.22
(0.2 PEG_{113} + 0.8 PEG_{45}) – $PHPMA_{400}$	53,500	1.95

Table S3. Summary of solution viscosities for various aqueous ammonium sulfate solutions at 20 °C.[3] These values were used for DLS analysis.

Concentration / M	Viscosity / mPa s
0.25	1.0414
0.5	1.1083
0.75	1.1684
1.0	1.2559
1.25	1.3043
1.5	1.4210
1.75	1.5663
2.0	1.6497
3.0	2.5303

References

1 M. J. Rymaruk, K. L. Thompson, M. J. Derry, N. J. Warren, L. P. D. Ratcliffe, C. N. Williams, S. L. Brown and S. P. Armes, *Nanoscale*, 2016, **8**, 14497–14506.

2 N. J. W. Penfold, Y. Ning, P. Verstraete, J. Smets and S. P. Armes, *Chem. Sci.*, 2016, **7**, 6894–6904.

3 R. C. Weast, *Handbook of Chemistry and Physics*, CRC Press, 66th edn., 1985.
